# Automatic brain-tumor diagnosis using cascaded deep convolutional neural networks with symmetric U-Net and asymmetric residual-blocks

**DOI:** 10.1038/s41598-024-59566-7

**Published:** 2024-04-25

**Authors:** Mahmoud Khaled Abd-Ellah, Ali Ismail Awad, Ashraf A. M. Khalaf, Amira Mofreh Ibraheem

**Affiliations:** 1 Faculty of Artificial Intelligence, Egyptian Russian University, Cairo, 11829 Egypt; 2 College of Information Technology, United Arab Emirates University, P.O. Box 15551, Al Ain, United Arab Emirates; 3 Faculty of Engineering, Al-Azhar University, P.O. Box 83513, Qena, Egypt; 4 Department of Electrical Engineering, Faculty of Engineering, Minia University, Minia, 61519 Egypt

**Keywords:** Biomedical engineering, Design, synthesis and processing, Magnetic resonance imaging, Computer science, Software

## Abstract

The use of various kinds of magnetic resonance imaging (MRI) techniques for examining brain tissue has increased significantly in recent years, and manual investigation of each of the resulting images can be a time-consuming task. This paper presents an automatic brain-tumor diagnosis system that uses a CNN for detection, classification, and segmentation of glioblastomas; the latter stage seeks to segment tumors inside glioma MRI images. The structure of the developed multi-unit system consists of two stages. The first stage is responsible for tumor detection and classification by categorizing brain MRI images into normal, high-grade glioma (glioblastoma), and low-grade glioma. The uniqueness of the proposed network lies in its use of different levels of features, including local and global paths. The second stage is responsible for tumor segmentation, and skip connections and residual units are used during this step. Using 1800 images extracted from the BraTS 2017 dataset, the detection and classification stage was found to achieve a maximum accuracy of 99%. The segmentation stage was then evaluated using the Dice score, specificity, and sensitivity. The results showed that the suggested deep-learning-based system ranks highest among a variety of different strategies reported in the literature.

## Introduction

A brain tumor is an abnormal growth of tissues that appears in the brain and can affect its function. The number of new brain-tumor cases in the United States in 2019 was 23,820, and there were an estimated 17,760 deaths from the condition in that year, according to the American Cancer Society’s Cancer Statistics Center^1,2^. Furthermore, the National Brain Tumor Foundation has announced that the number of individuals in advanced nations who die because of a cerebral tumor in recent decades has increased by 300%^3^. Gliomas are the best-known kind of brain tumor, and these can be divided into high- and low-grade gliomas (HGGs and LGGs); HGGs tend to result in a 2-year life expectancy, whereas people with LGGs can have a life expectancy of several years or more. The most common brain-tumor treatment methods that are applied to reduce tumor growth are surgery, chemotherapy, and radiotherapy^4^.

Magnetic resonance imaging (MRI) is a high-quality imaging technique that can provide substantial information about brain tissue; as such, it has been widely used for automatic tumor diagnosis^5^. MRI can provide highly detailed images of the features of brain tumors and, as a result, can result in additional therapeutic options becoming available to a patient. The technique can also give information about the physiology, metabolism, and hemodynamics of certain tumors. MRI scans are ideal for soft-tissue imaging. Because of the prevalence of brain tumors, a large amount of brain-tumor MRI data is generated; developing an automated brain-tumor diagnostic system with acceptable performance is thus critical^6^. The use of a computer-aided diagnosis (CADx) system is essential for detecting brain tumors quickly and without the need for human interaction. The treatment for a brain tumor will vary according on the type of tumor and its size and location. Brain-tumor classification and segmentation are thus crucial tasks for diagnosing tumors, determining treatment decisions, and increasing the likelihood of recovery^7,8^.

Automatic brain-tumor detection using MRI scans can significantly improve diagnosis, therapy, and growth-rate prediction. Automatic tumor diagnosis includes a pipeline of three processes: detection, segmentation, and classification. Detection aims to classify MRI images with respect to the presence or absence of a tumor into abnormal and normal images, respectively^9^. Segmentation aims to recognize the tumor zone and delineate the boundaries of its regions: necrotic tissue, active tumor tissue, and edema (growth near the tumor). Classification aims to categorize MRI images of gliomas into HGGs and LGGs^10^.

Segmentation is realizable by identifying regions that appear different when compared to ordinary tissues. While some tumors—for example, meningiomas—are easily segmented, others—such as gliomas—are fundamentally harder to segment. These tumors tend to have edema and extended limb-like structures, are very often diffused, and provide limited image contrast, which makes the division procedure difficult. Their boundaries are frequently hazy and difficult to distinguish from healthy tissues. Moreover, they can appear in any region of the brain and can have a range of different shapes and sizes^11^. As a result, the problem of brain-tumor diagnosis can be seen as a difficult image-classification task.

In general, machine learning, and particularly deep-learning algorithms, can greatly assist with the detection, segmentation, classification, and registration of brain tumors. Recently, deep-learning techniques have gained greater research attention^12,13^. Deep-learning methods can be trained in their convolution layers using either unsupervised or supervised training methods^14^. The findings of recent studies have shown that deep-learning approaches outperform traditional learning methods in brain-tumor diagnosis^15^, and several systems have been designed for brain-tumor detection, classification, and segmentation based on deep learning.

Because of the high incidence of brain tumors, a vast amount of MRI data has been collected. Gliomas, with their irregular shapes and ambiguous boundaries, are the most challenging tumors to identify. The detection and classification of brain tumors are critical steps that are dependent on a diagnosing physician’s expertise and knowledge. Although various studies have focused on the use of deep-learning approaches to brain-tumor diagnosis, no comprehensive system for automatic tumor detection, classification, and segmentation is currently available, and a complete technique for automatic brain-tumor diagnosis has not yet been published in the literature. Furthermore, accurately integrating tumor segmentation, classification, and detection inside a single system is still an open problem. The presence of the brain-tumor detection and classification phases before tumor segmentation in a single system will result in normal images being excluded from the segmentation phase. This allows for real-time deployment of automatic tumor-diagnosis systems, saving time and computing power that would otherwise be used in attempting to identify tumors in normal images. An intelligent strategy for detecting and classifying brain tumors is essential for supporting clinicians, and it could be a beneficial tool in hospital emergency rooms when examining the MRI scans of patients, since it would allow for speedier diagnosis. For example, such a system could help doctors by examining MRI images before processing to identify whether they are normal or contain an HGG or LGG. Once an image has been automatically identified as abnormal, a physician can easily detect and segment the brain tumor to estimate its size using the segmentation stage.

In this study, the aforementioned challenges have been addressed by developing an automatic CADx system for brain-tumor detection, classification, and segmentation. In the first stage, we introduce a convolutional neural network (CNN) architecture for detection and classification. In the second stage, a CNN architecture for segmentation is introduced. These systems use state-of-the-art CNN architecture and training procedures, including the U-Net, residual units, batch normalization (BN), dropout regularization, the parametric rectified linear unit (PReLU), and skip connections. Additionally, both the contexts and local shapes of tumors are taken into consideration. The proposed technique overcomes the issue of performing pixel arrangement without considering the local dependencies of labels.

The proposed system’s accuracy was assessed using MRI images derived from a brain-tumor segmentation database. In addition to improving the overall structure of the system, the major research contributions can be summarized as follows.

1. A new and fully automatic brain-tumor diagnosis structure has been built by combining the detection and classification phases prior to segmentation, saving time and computing power. This can support clinicians and be a beneficial tool for real-time deployment in hospital emergency rooms.
2. A new detection and classification CNN has been constructed by combining two parallel paths, using residual units, and selecting the best values of convolutional-layer filters to help extract more features.
3. The proposed segmentation model has been designed using two asymmetric parallel paths with two U-Net architectures in series and a residual encoder and decoder for brain-tumor segmentation.
4. Both local and global features are considered, including local details of the brain, to improve the detection, classification, and segmentation performances.
5. The use of feature fusion further enhances the extraction of multi-scale features.
6. The proposed brain-tumor diagnosis system was optimized in several respects, including in terms of the accuracy and speed of diagnosis.
7. Comparisons were made among the results of different experiments to obtain a model with the highest accuracy and Dice score, resulting in a model for brain-tumor detection, classification, and segmentation that is superior to any previously published.

The remainder of this paper is structured as follows. The next section Related work is dedicated to exploring works related to the detection, classification, and segmentation of brain tumors. The structures of the proposed system are then presented in Materials and Methods. Subsequently, the implementation and performance-evaluation processes are described in Simulations and Evaluation Criteria. A discussion of the outcomes and research findings is then given in Results and Discussion. Finally, the conclusions of the research are presented in Conclusions and Future Work.

## Related work

Over recent decades, the diagnosis of brain tumors has gained considerable research interest, and many diagnosis techniques have been introduced. The performance of tumor diagnosis has been improved by applying several different automatic brain-tumor detection techniques. In these approaches, machine learning is crucial for the identification, classification, and segmentation of brain tumors, and several machine-learning algorithms have recently been developed to this end as shown in Table 1.

El-Dahshan et al.^16^ used the discrete wavelet transform (DWT) as a feature extractor, principal component analysis (PCA) for feature minimization and selection, and a three-layer artificial neural network (ANN) for the actual tumor detection. Abd-Ellah et al.^3^ combined morphological filters, the DWT, PCA, and a kernel support vector machine (KSVM) for brain-tumor detection from MRI images. Using a twofold classifier, researchers were able to categorize a picture as benign (noncancerous) or malignant (cancerous)^17^. Using the DWT as a feature extractor, Zhang et al.^18^ were able to discover brain tumors in MRI images. The features were reduced from 65,536 to 1024 using a three-level decomposition with Haar wavelets. The reduced characteristics were then sent into a back-propagation neural-network classifier. Devasena et al.^19^ demonstrated a CADx system for MRI-based tumor diagnosis that employs a hybrid abnormal detection algorithm.

Patil et al.^20^ applied a probabilistic neural network (PNN) for extracting features to detect brain tumors; they applied the *k*-nearest neighbors (*k*-NN) algorithm, an ANN, and an SVM to detect and recognize various types of tumor^21^. Goswami et al.^22^ also proposed a brain-tumor classification system using MRI images. Noise filtering, edge detection, and histogram equalization are used in the preprocessing stage of their system; then, independent component analysis is applied for feature extraction, and classification is performed using a self-organized map. Deepa and Devi^23^ used a combination of feature extraction, segmentation, and tumor classification to diagnose brain tumors. In their approach, optimal texture characteristics are extracted using statistical features, and a radial basis function neural network and a back-propagation neural network are used in the segmentation and classification stages, respectively.

Sarith et al.^24^ presented a technique for detecting brain tumors from MRI images that uses wavelet entropy-based spiderweb plots for feature extraction and a PNN for tumor identification. Yang et al.^25^ suggested a technique using 2D-DWT and Haar-wavelet feature extraction for early brain-tumor detection from MRI images with a KSVM as a classifier. Kalbkhani et al.^26^ also used 2D-DWT and modeled the sub-bands of detail coefficients using a generalized autoregressive conditional heteroscedasticity model. Linear discriminant analysis was used to extract 61,440 features, which were then reduced to 24 using PCA; the *k*-NN and SVM methods were employed for the actual tumor detection. Mudda et al.’s^27^ main goal was to detect whether a brain has a tumor or is healthy. Gray-level run-length matrix (GLRLM) texture characteristics were employed to extract the features for efficient brain-tumor diagnosis using neural-network methods. In 2023, Asiri et al.^28^ employed six machine-learning methods for brain-tumor detection: SVM, neural networks, random forest (RF), CN2 rule induction (CN2), naive Bayes (NB), and decision trees. Achieving 95.3% accuracy, it was found that SVM surpassed other methods.

Abd-Ellah et al.^5^ created a deep-learning technique for detecting and classifying brain tumors from MRI images. As a feature extractor, they employed the AlexNet CNN, and at the classification step, an error-correcting output codes SVM was applied. For feature extraction and selection, Heba et al.^29^ used the DWT was used with PCA. A seven-layer deep neural network (DNN) was used to classify the retrieved characteristics. Tazin et al.^30^ employed a CNN and a transfer-learning technique to determine whether or not a brain tumor was present in X-ray images; VGG19, InceptionV3, and MobileNetV2 were used for deep feature extraction. MobileNetV2 was found to be 92% accurate, InceptionV3 was 91% accurate, and VGG19 was 88% accurate. Alsaif et al.^31^ applied multiple CNN models based on data augmentation to identify brain tumors using MRI images. VGG16, VGG19, ResNet-50, ResNet-101, Inception-V3, and DenseNet121 were applied to distinguish between normal samples and those containing a tumor. The best accuracy, found with VGG16, was established as 96%.

Techniques for semi-automatic and automated brain-tumor segmentation can be broadly split into discriminative and generative models^32^. Brain-tumor segmentation approaches based on generative models require previous knowledge of the shape, size, and appearance of both tumor and normal tissues, which may be obtained in probabilistic picture atlases^33,34^, in which the tumor segmentation is modeled as a detection problem with a probabilistic image. The generative framework of tumor segmentation using outlier detection through MRI is represented in Ref.^35^. Based on a probabilistic technique, Prastawa et al.^36^ initialized active contours on a brain atlas and iterated until the probability was below a specific threshold. Different methods based on active contours have been presented^37,38^. Because aligning a large brain tumor onto a model is a challenging task, some techniques apply registration alongside tumor segmentation, as in Ref.^39^.

Brain-tumor segmentation methods based on discriminative models use the extraction of image features to classify image voxels as normal or tumor tissues. The performance of a discriminative model depends on the extracted features and classification techniques. Different image features have been considered in brain-tumor-segmentation techniques, including image textures^40,41^, local histograms^42^, alignment-based features such as symmetry analysis, region-shape differences, inter-image gradients, tensor eigenvalues^43^, raw-input pixel values^44,45^, and discriminative-learning techniques such as decision forests^42,43^ and SVMs^46,47^.

Techniques based on deep learning have arisen as an effective alternative to traditional machine learning, as they have impressive capacity to learn discriminative features, outperforming models using pre-defined and hand-crafted features. More recently, deep-learning techniques have achieved success in general image-analysis studies, including object detection^48^, image classification^49^, and semantic segmentation^50–52^, and this success has led the approach to be applied to brain-tumor segmentation.

Specifically, CNNs were used in the BraTS 2014 challenge as a promising approach to segmenting brain tumors^53–55^. Additional brain-tumor-segmentation techniques using deep learning were introduced for BraTS 2015, and various deep-learning methods were presented, such as stacked denoising autoencoders, convolutional restricted Boltzmann machines, and CNNs^56–60^. Deep-learning brain-tumor segmentation methods that build upon CNNs have been found to achieve the best performance. In particular, both 2D-CNNs^4,53,54,58–63^ and 3D-CNNs^55,64,65^ have been adopted to develop brain-tumor-segmentation techniques.

Wang et al.^66^ presented a modality-pairing learning approach for segmenting brain tumors. Two parallel branches were created to leverage the properties of various modalities, and several layer interconnections were used to capture complicated interactions and extract a wealth of information. To reduce the prediction variation between the branches, they applied a consistency loss. Furthermore, a learning-rate warmup technique was used to deal with the problem of training instability. The authors of Ref.^67^ introduced a cross-modality deep-feature-learning approach for segmenting brain tumors. This system was made up of two procedures: cross-modality feature fusion (CMFF) and cross-modality feature transition (CMFT); they used the BraTS 2017 and 2018 datasets. Remya et al.^68^ demonstrated tumor segmentation based on a fuzzy c-means (FCM) technique and an improved noise-filtering computation. They updated the noise-filtering computation to obtain the right tumor region, and the execution was improved by upgrading the threshold function. Following filtering, segmentation was performed using Otsu’s method and the FCM approach. The Jaccard and Dice coefficients were 0.5304 and 0.6893, respectively, according to the findings.

Most of these methods were trained using small image patches to classify images into various classes. The classified patches are used to label centers for achieving segmentation. However, most of the methods used assume that a voxel label is distinct, and they do not consider spatial consistency and appearance. Havaei et al.^4^ constructed a cascaded two-pathway architecture, and they provided the probabilities of pixel-wise segmentation results acquired from the first CNN architecture as an extra input to the second CNN to take into account the local dependencies of labels. Additionally, the spatial consistency and appearance can be taken into consideration, as in Ref.^64^, and conditional random fields (CRFs) and Markov random fields can be combined with CNN segmentation methods as a post-processing step or created as neural networks according to Refs.^51,52^. Myronenko^69^ proposed a semantic segmentation network to segment tumor subregions from 3D MRI images using an encoder–decoder architecture. In this system, the autoencoder branch reconstructs the input image and the decoder regularizes and imposes additional constraints on its layers. This approach won first place in the BraTS 2018 challenge.

Fritscher et al. presented a network architecture based on 3D input patches to three convolutional pathways in the coronal, sagittal, and axial views, which are merged by fully connected layers^70^. Pereira et al.^61^ proposed a tumor-segmentation technique that applies intensity normalization in the preprocessing stage. Later, they presented a hierarchical study based on a fully convolutional network (FCN) and histograms using MRI images^62^. Zhao et al.^71^ combined CRFs and an FCN to segment brain tumors in three training stages. Dong et al.^72^ used a U-Net approach with comprehensive data augmentation to propose a fully automatic 2D method, and a 3D U-Net network was applied for tumor segmentation, providing shortcut connections between the upsampling and downsampling paths. Various additional approaches for detecting, segmenting, and classifying brain tumors are outlined in Ref.^73^.

**Table 1:** Comparison between different classification and segmentation literature.

Type	References	Year	Used Technique	Dataset	AC/Dice
Classification	Pan et al.^74^	2015	CNN	BraTS 2014	73.33
	Abd-Ellah et al.^3^	2016	DWT, PCA, and KSVM	Collected Data	66.96
	Ye et al.^75^	2017	3D CNN	BraTS 2015	82.1
	Ge et al.^76^	2018	3D CNN	BraTS 2017	89.47
	Heba et al.^29^	2018	DNN	Collected Data	96.97
	Sultan et al.^77^	2019	CNN	Collected Data	95.81
	Anaraki et al.^78^	2019	DNN	Collected Data	96.50
	Abd-Ellah et al.^79^	2019	PDCNN	BraTS 2017	97.44
	Tazin et al.^30^	2021	CNN	Collected Data	92.00
	Mudda et al.^27^	2022	GLRLM, CSLBP, and ANN	Collected Data	94.00
	Alsaif et al.^31^	2022	CNN	Kaggle	96.00
	Asiri et al.^28^	2023	RF, NB, and SVM	Kaggle	90.7
Segmentation	Zhao et al.^71^	2016	FCN and CRF	BraTS 2013	0.87
	Pereira et al.^61^	2016	CNN	BraTS 2013	0.84
	Pereira et al.^62^	2017	FCN	BraTS 2013	0.85
	Mohamad et al.^4^	2017	DNN	BraTS 2013	0.88
	Kamnitsas et al.^64^	2017	3D CNNs	BraTS 2015	0.85
	Zhao et al.^63^	2018	FCNs and CRFs	BraTS 2016	0.88
	Abd-Ellah et al.^2^	2019	TPUAR-Net	BraTS 2017	0.89
	Zhang et al.^67^	2021	CMFT and CMFF	BraTS 2018	0.90
	Wang et al.^66^	2021	3D U-Net	BraTS 2020	0.89
	Remya et al.^68^	2022	Otsu and FCM	BraTS 2015	0.72

**Figure 1:**
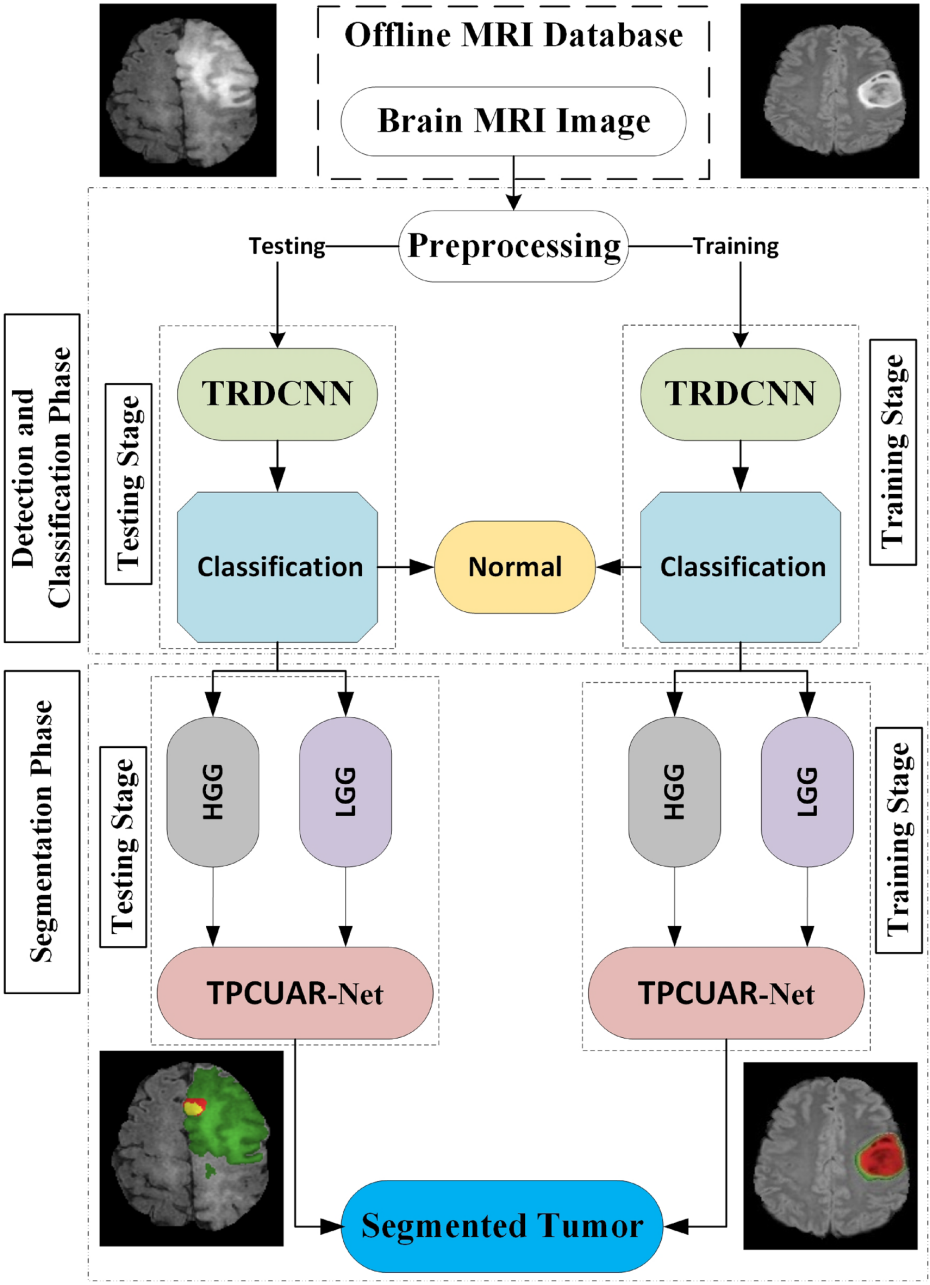
Flowchart depicting the two phases and illustrating the two distinct types of CNN employed. The two-pathway with residual-based deep convolutional neural network (TRDCNN) architecture is used in the detection and classification phases, and two parallel cascaded U-Nets with an asymmetric residual (TPCUAR-Net) is employed in the segmentation phase.

## Materials and methods

As outlined in the previous section, deep-learning methods have attracted considerable research interest because they provide the ability to process a huge number of MRI images with great efficiency^5^. The proposed diagnosis system has two stages. The first stage focuses on tumor detection and classification by categorizing images into normal, HGG, and LGG. The second stage converts tumor segmentation into a classification problem. Figure 1 shows a flowchart of the developed system. MRI images are provided to the architecture’s input, and preprocessing is applied. Then, a CNN is used to extract and classify the features of each MRI image to determine if it is normal or abnormal. To achieve accurate results, the parallel architecture of the CNN is first trained, and a second CNN is applied in the second-stage segmentation. The system was evaluated using the Reference Image Database to Evaluate Therapy Response (RIDER) and the BraTS 2017 dataset as standard reference MRI datasets.

### Preprocessing stage

Image preprocessing is vital in diagnostic tasks, as it enhances the quality of images and prepares them for precise and efficient diagnosis. The BraTS 2017 database has 3D MRI volumes with various spacings in the three dimensions. In the present work, detection, classification, and segmentation were applied to slices with various image modalities (FLAIR, T2, T1, and T1C). In the preprocessing stage of the proposed approach, each volume is cropped to remove the unwanted background, which saves computational power. Then, each slice is normalized, excluding the ground truth, by subtracting the mean and dividing by the standard deviation. After that, the top and bottom pixel intensity are clipped by one percent. All normal slices are ignored in the segmentation stage. The axes are swapped to represent the modalities in axis 0 and the slice in axis 1. All slices are randomly shuffled.

**Figure 2:**
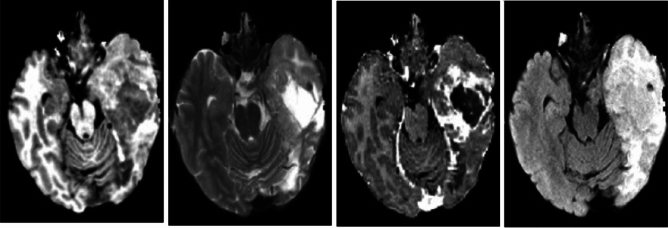
Samples of preprocessed images.

In the first stage, the slices are resized to $256 \times 256$ due to the variation in MRI slice size. Therefore, in the segmentation stage, patches of $128 \times 128$ size are generated. Patches are randomly shuffled and randomly selected. By rescaling or resizing the images to a standardized format, we can ensure consistent performance of the diagnosis algorithm across different images. Moreover, resizing can also reduce computational complexity. Data augmentation can play a vital role in diagnostic tasks by generating additional training samples through the application of various transformations to an existing dataset. By introducing variations in the training data, data augmentation enhances a model’s resilience and ability to handle a wide range of scenarios. Samples of enhanced images are shown in Fig. 2.

### The main CNN architectures

#### Convolutional layers

The convolutional layers are the fundamental building blocks of the CNN architecture; they provide feature maps from input maps, except for the first convolutional layer, for which the input is taken directly from an input image. The feature computation $M_{s}$ is calculated as in Eq. (1):

1 $$\begin{aligned} M_{s}= b_{s} + \sum _{r} W_{sr} * X_{r} \end{aligned}$$

where $W_sr$ denotes the input channel sub-kernel, $X_r$ is the *r*th input channel, and $b_s$ is a bias term. The convolutional layers can separately learn the biases and weights of the feature map, extending the data-driven, customized, and task-specific dense feature extractors. As a result, a nonlinear activation function is applied.

#### Rectified linear unit (ReLU) and parametric ReLU (PReLU) layers

ReLU activation functions are frequently used for the hidden layers. When the input is greater than 0, the output *y* is the same as the input *x*; otherwise, the output is neglected as in Eq. (2):

2 $$\begin{aligned} f{y}= \max (x,0) \end{aligned}$$

PReLU offers a small number of parameters $\alpha$, which increases accuracy^80^ as in Eq. (3):

3 $$\begin{aligned} f{y}= \max (x,0) + \alpha \min (x,0) \end{aligned}$$

#### Max-pooling layers

Max-pooling minimizes the input dimension by down-sampling; the number of learned parameters is thus decreased, reducing cost, improving performance, and overcoming the problem of overfitting. A non-overlapping max filter is employed to subregions, taking $N \times N$ regions and providing a single value^5^.

#### Residual blocks

Residual blocks have skip connections that enable information to be transmitted both forward and backward directly between layers as in Eqs. (4)–(5):

4 $$\begin{aligned} y_{i}= & {} h(m_{i})+f(m_{i},W_{i}) \end{aligned}$$

5 $$\begin{aligned} m_{i+1}= & {} f(y_{i}) \end{aligned}$$

where $m_{i}$ is the *i*th input and $m_{i+1}$ is the *i*th output, $W_{i}$ is the set of weights, $h(m_{i})=m_{i}$ is known as attaching an identity skip connection, and $m_{i+1}=y_{i}$ when *f* is an identity^81^.

There is a stack of various layers in residual blocks: a convolutional layer, PReLU, and BN, which is used to normalize the input. Further, PReLU is again attached and followed by a convolution layer, and these layers are repeated. The input and the output are summed, generating a direct connection. The residual decoding block includes a convolution layer in the direct route, as shown in the bottom left of Fig. 5.

#### Regularization and loss function

The cross-entropy loss is applied to test the performance of the classification stage. This is developed as the predicted label probability as in Eq. (6):

6 $$\begin{aligned} L_{\textrm{ce}}(H, \hat{H})=H\log \left( \dfrac{H}{\hat{H}}\right) +(1-H)\log \left( \dfrac{1-H}{1-\hat{H}}\right) , \end{aligned}$$

where *H* is the desired output, $L_{\textrm{ce}}(H, \hat{H})$ is the cross-entropy error, and $\hat{H}$ is the predicted output^82^.

BN is used for regularizing the provided values and eliminating nonlinearities as in Eq. (7):

7 $$\begin{aligned} \hat{S}= \dfrac{S-\textrm{E}[S]}{\sqrt{\textrm{Var}[S] }} \end{aligned}$$

where *S*, $\hat{S}$, $\textrm{Var}[S]$, and *E*[*S*] are the input layer, the normalized activations, the unbiased variance estimate, and the expectation value, respectively^83^. BN allows the CNN to be trained with stable gradients, smoothing the optimization plane, making weight initialization easier, and reaching optimal values more rapidly with more viable activation functions. The weighted loss and regularization function reduces the problem of becoming stuck in local minima and enhances the performance of the model. Another regularization technique is dropout, which randomly ignores selected neurons during training and stops weights from updating^62^.

**Figure 3:**
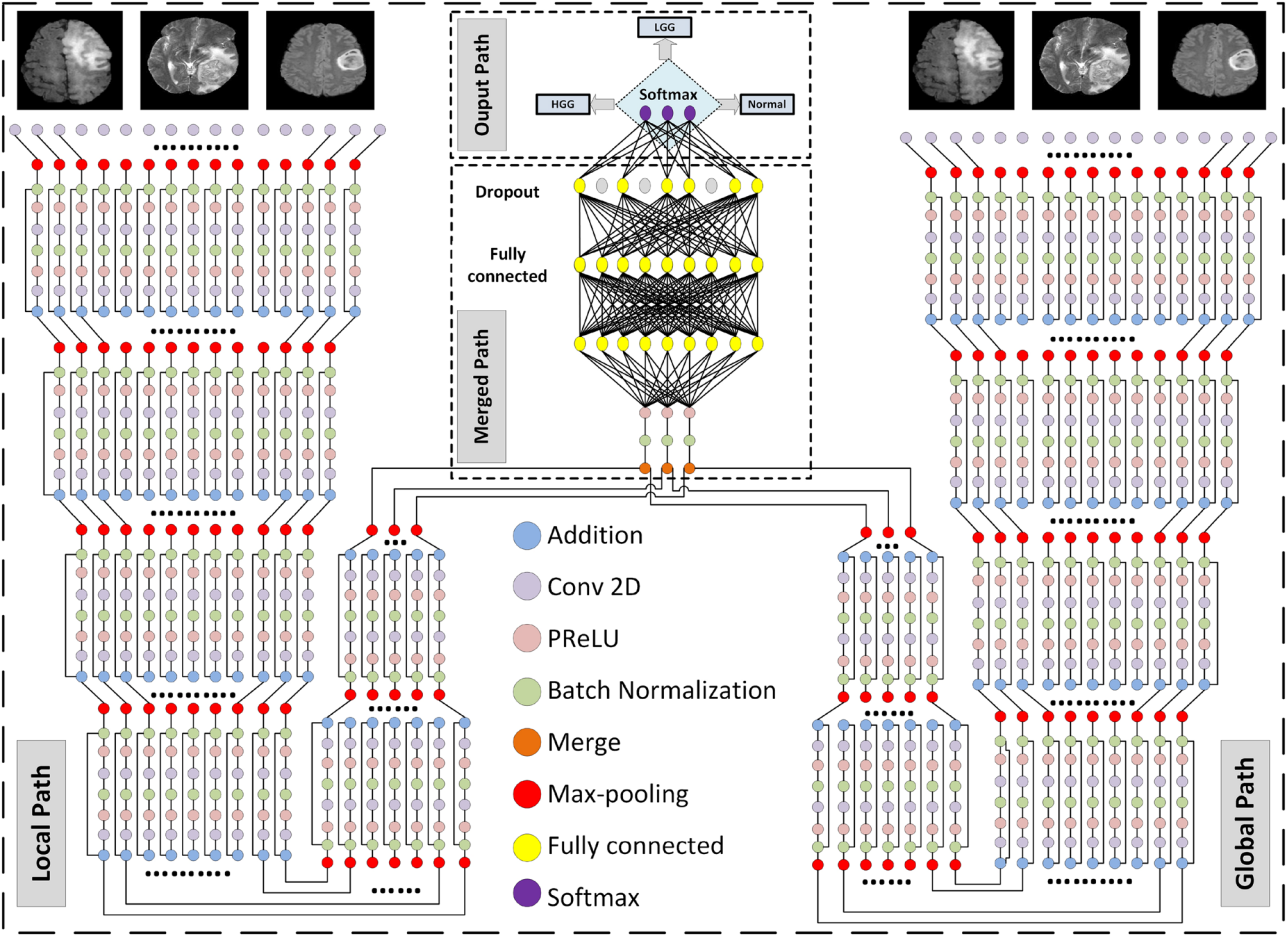
Proposed TRDCNN structure for tumor detection and classification.

#### Softmax layer

Softmax is a widely used layer in CNNs for multi-class classification tasks. It is normally positioned at the end of the network and generates a probability distribution over the classes. The softmax layer normalizes the outputs of the preceding layer to indicate the probability of each class. The softmax function calculates the probability of each class by multiplying the input scores and dividing them by the sum of all exponentiated values as in Eq. (8):

8 $$\begin{aligned} Y_{j}(x)= \dfrac{\exp {x_{j}}}{\sum _{i}\exp {x_{i}} } \end{aligned}$$

where $Y_{j}(x)$ is the probability of the input instance in class *j*, and $x_{j}$ is the *j*th element of the vector $x_{i}$.

### The first stage: the proposed brain glioma detection and classification architecture

In this work, we examined different architectures by applying feature-map concatenation between different numbers of layers when composing CNNs. This operation produced various architecture designs with different computational routes. We now present the best architecture that was found during this exploration process.

#### Two-pathway with residual-based deep convolutional neural network architecture (TRDCNN)

Detection and classification of glioblastomas are conducted using the proposed TRDCNN. A schematic of the proposed structure is provided in Fig. 3. The input is MRI images; as noted, these are subjected to preprocessing to decrease the calculation complexity and to speed up processing. The proposed structure comprises four paths: the global, local, merged, and output paths. The local and global paths have their own responsibilities in feature extraction; they have one of each of a convolutional (CONV), ReLU, and max-pooling layer, followed by six stages of residual blocks with max-pooling. The first CONV layer uses a small filter of $5 \times 5$ pixels in the local path and a large filter of $12 \times 12$ pixels in the global path. The global and local paths are connected in parallel, and they are then combined into a merged path that includes BN, ReLU, and fully connected layers, followed by a dropout layer. The final path is the output path, which contains a classification layer with a softmax function; this classifies features into normal and glioblastoma (HGG or LGG) images.

### The second stage: brain-tumor segmentation architectures

A CNN usually has a huge number of parameters; patch-based training can be applied to train a DCNN with enough samples^58^. As such, the segmentation problem can be handled as a classification problem. Image patches are selected regions that describe a central pixel, and they are labeled with the label of their center pixel. Millions of image patches can be generated to train a CNN. In the testing process, all the extracted patches are classified by the trained network, which then makes up a segmented image. However, CNN techniques segment images slice by slice^4^. Furthermore, the locations of training patches and the number for each class are controlled by changing the patch-selection scheme.

**Figure 4:**
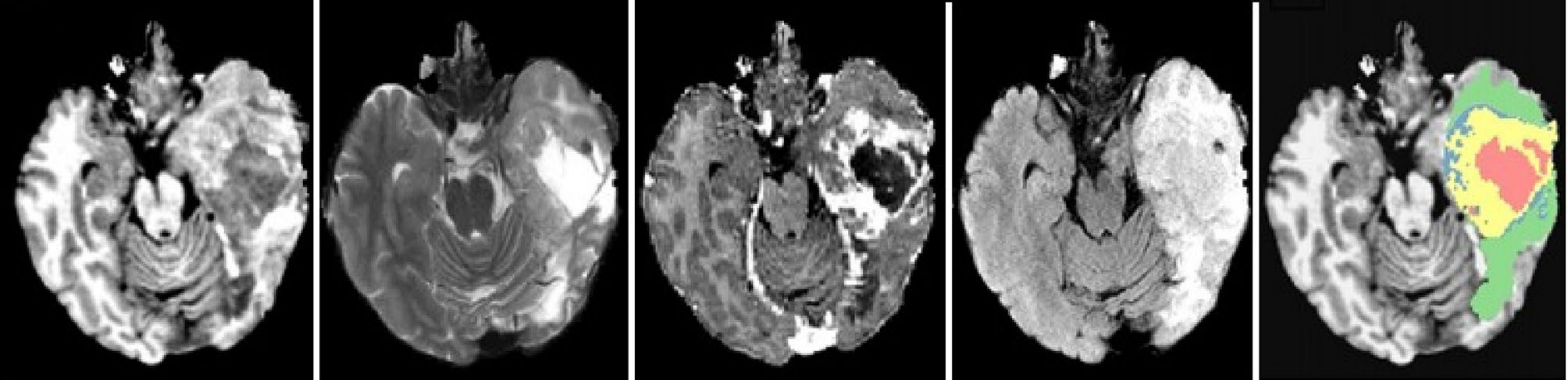
Left to right: the four MRI modalities—T1, T2, T1-C, and FLAIR—and the ground truth. The latter shows regions as follows: enhancing tumor , necrosis , edema , and non-enhancing tumor . This is taken from the authors’ own work (Ref.^2^).

#### Two parallel cascaded U-Nets with an asymmetric residual (TPCUAR-Net)

**Figure 5:**
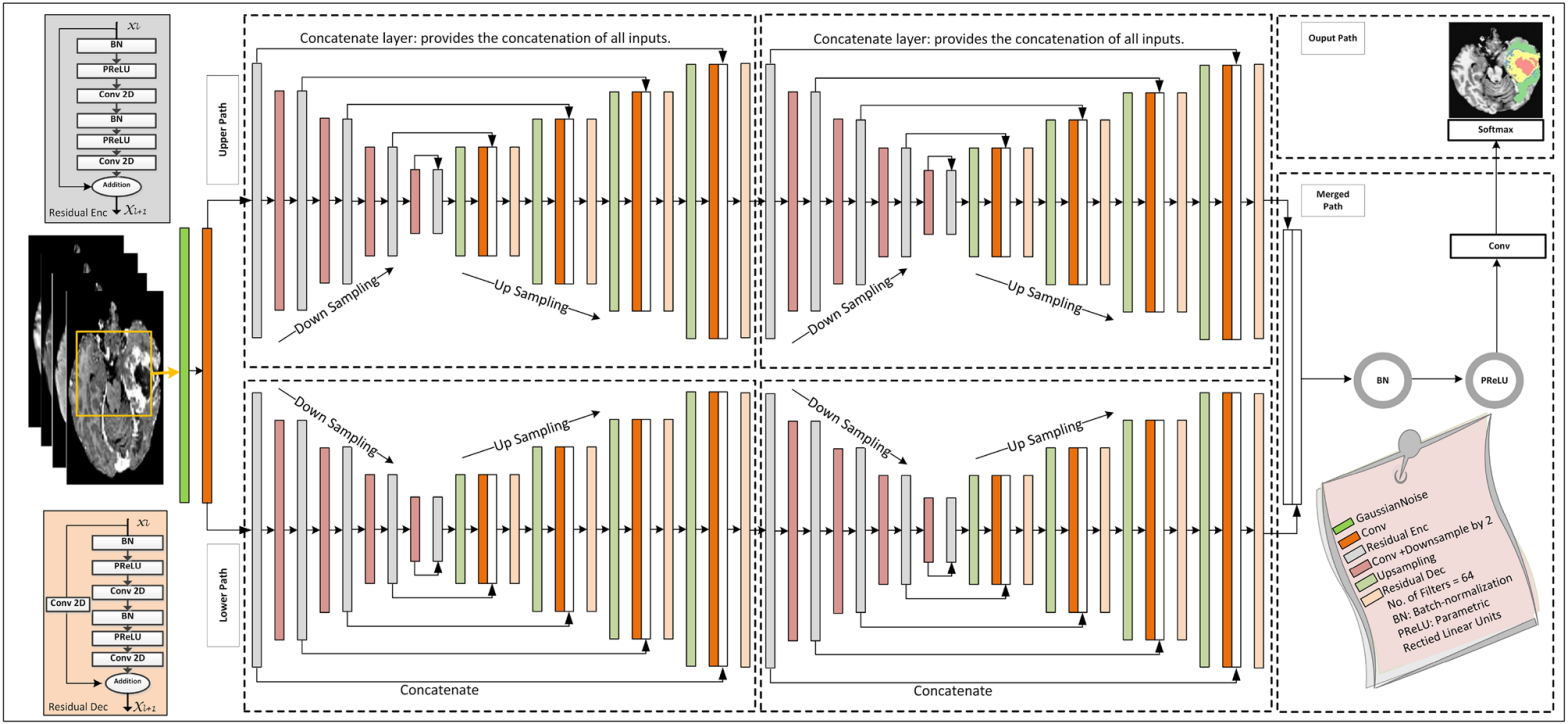
TPCUAR-Net architecture for brain-tumor segmentation.

Figure 5 shows the TPCUAR-Net architecture. The input to this structure is an MRI image resulting from merging five different MRI modalities (Fig. 4). The noise is reduced and image quality is improved by preprocessing. There are four paths: the structure, upper, lower, merged, and output paths. The upper and lower paths each comprise two series U-Nets with various depths, which are used in feature extraction to elicit the local and global features. These have up- and down-sampling processes, and two different residual blocks called Residual Enc., and Residual Dec., as in Fig. 5. The convolutional layer has a two-step stride in the down-sampling path and a one-step stride in the up-sampling path. The merged path combines the upper and lower paths in parallel form; it comprises concatenation, BN, PReLU, and convolutional layers. The concatenation layer receives a group of inputs with the same shape and combines them, providing one path. The output path produces an image segmented with a softmax function.

## Simulations and evaluation criteria

This section describes simulations conducted to evaluate the proposed system using several different metrics. The approach was developed using Jupyter Notebook, and the Keras and TensorFlow toolkits were used to code the suggested architecture. The computer used for this task has a 3.2 GHz Intel Core i7 processor, 24 GB of RAM, and was running Windows 7 64-bit as its operating system.

**Figure 6:**
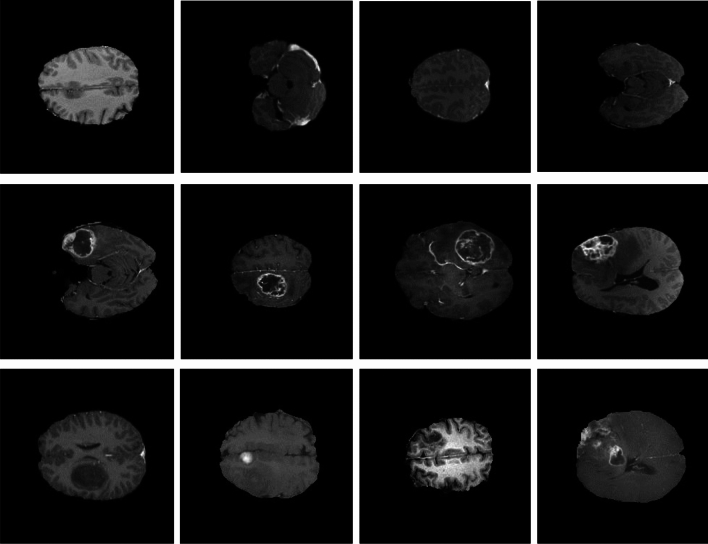
Sample images used for the experimental work. From top to bottom, the rows show normal, HGG, and LGG brain images.

### Imaging data

The images used in this manuscript were extracted from the BraTS 2017 dataset. This provides images from 285 glioma patients, 210 with HGG and 75 with LGG. The data were scanned using different clinical protocols and scanners from different institutions, including Heidelberg University, the University of Alabama, the University of Bern, the National Institutes of Health, the University of Debrecen, and The Center for Biomedical Image Computing and Analytics. Multimodal scans are available for each patient, including T1, T1ce, T2, and FLAIR volumes^32^.

The data used during BraTS 2014, 2015, and 2016 (from The Cancer Imaging Archive) were discarded, as their ground-truth labels were generated from the highest-ranking methods during BraTS 2012 and 2013. However, expert radiologists were included in BraTS 2017, which lead us to use these data instead of other BraTS datasets. The training and testing datasets are listed in Table 2, and Fig. 6 presents a sample of the MRI images used in the investigation.

**Table 2:** Training and testing dataset distributions.

		Training set		Testing set	
Classification	Normal	450		150	
	HGG	450		150	
	LGG	450		150	
	Total images	1350		450	
Segmentation	**Type**	**LG**	**HG**	**LG**	**HG**
	No. of patients	40	60	20	50
	3D volume	160	240	80	200
	2D slice	24800	37200	12400	31000
	Total images	62,000		43,400	

### Training and testing

The proposed model was trained using the image database for 100 epochs using the SGD optimizer and a learning rate of 0.08. The batch size was 16, and the error loss was calculated using the square of the mean. All details of training and testing parameters are listed in Table 3. Typically, a grid or random search is performed in hyperparameter space, followed by training the proposed models for a predetermined number of epochs for each hyperparameter choice. The best hyperparameter is then determined by selecting the value with the highest validation accuracy. The parameters are chosen based on a trial-and-error method.

**Table 3:** Training parameters.

Type	Classification	Segmentation
Slice size	$256 \times 256$	$128 \times 128$
Pooling operations	Max-pooling	Max-pooling
Features after first conv.	290,400	524,288
Optimizer	SGD	SGD
Learning rate	0.001	0.08
Epochs	100	100
Patch size	128	32
Momentum	0.9	0.9
Decay	0.000005	0.000005

### Evaluation metrics

#### Tumor detection and classification

The metrics used for evaluation were as follows: sensitivity (SV), which indicates the proportion of correctly classified positives; specificity (SP), which is the proportion of correctly classified negatives; and accuracy (AC), which represents the proportion of both true negatives and true positives. These are calculated as in Eqs. (9)–(11), respectively^5^:

9 $$\begin{aligned} {{\textbf {Sensitivity (SV)}}}= & {} \frac{\sigma }{\sigma + \Phi } \end{aligned}$$

10 $$\begin{aligned} {{\textbf {Specificity (SP)}}}= & {} \frac{\eta }{\eta + \Psi } \end{aligned}$$

11 $$\begin{aligned} {{\textbf {Accuracy (AC)}}}= & {} \frac{\sigma + \eta }{\sigma + \eta + \Phi + \Psi } \end{aligned}$$

where $\sigma$ is the number of true positives, $\Phi$ is the number of false negatives, $\eta$ is the number of true negatives, and $\Psi$ is the number of false positives. The intersection over Union (IoU) is a popular metric to measure localization accuracy for binary classification; it is calculated as in Eq. (12)^84^:

12 $$\begin{aligned} {{\textbf {Intersection over Union (IoU)}}}= \frac{\sigma }{\sigma + \Phi + \Psi } \end{aligned}$$

#### Tumor segmentation

The system’s performance on the test set was determined by contrasting the prediction output with the ground truth supplied by knowledgeable radiologists. There are three distinct types into which tumor structure are categorized; the primary reason for this is practical clinical applications. These are: a full tumor (which includes all types), a core tumor (which includes all types save edema, and an augmenting tumor (which includes enhancing). The Dice coefficient, sensitivity, and specificity were computed for each tumor location as in Eqs. (13)–(15):

13 $$\begin{aligned} {{\textbf {Dice}}}= & {} \dfrac{|O\cap G|}{(|O|+|G|)/2} \end{aligned}$$

14 $$\begin{aligned} {{\textbf {Sensitivity}}}= & {} \dfrac{|O\cap G|}{|G|} \end{aligned}$$

15 $$\begin{aligned} {{\textbf {Specificity}}}= & {} \dfrac{|O_{0}\cap G_{0}|}{|G_{0}|} \end{aligned}$$

where *O* is positive segmented regions, $O_0$ is negative segmented regions, *G* is the actual ground truth, and $G_0$ is falsely identified regions. The intersection point between *O* and *G* is $|O\cap G|$^4^. Another popular performance metric for determining the separation between two point sets is the average Hausdorff distance^85^ as in Eq. (16):

16 $$\begin{aligned} {\textbf {Hausdorff}}(x,y) = \left( \dfrac{1}{x}\sum _{x\in X}\min _{y\in Y}d(x,y)+\dfrac{1}{y}\sum _{y\in Y}\min _{x\in X}d(x,y)\right) \end{aligned}$$

## Results and discussion

### Tumor detection accuracy analysis

**Figure 7:**
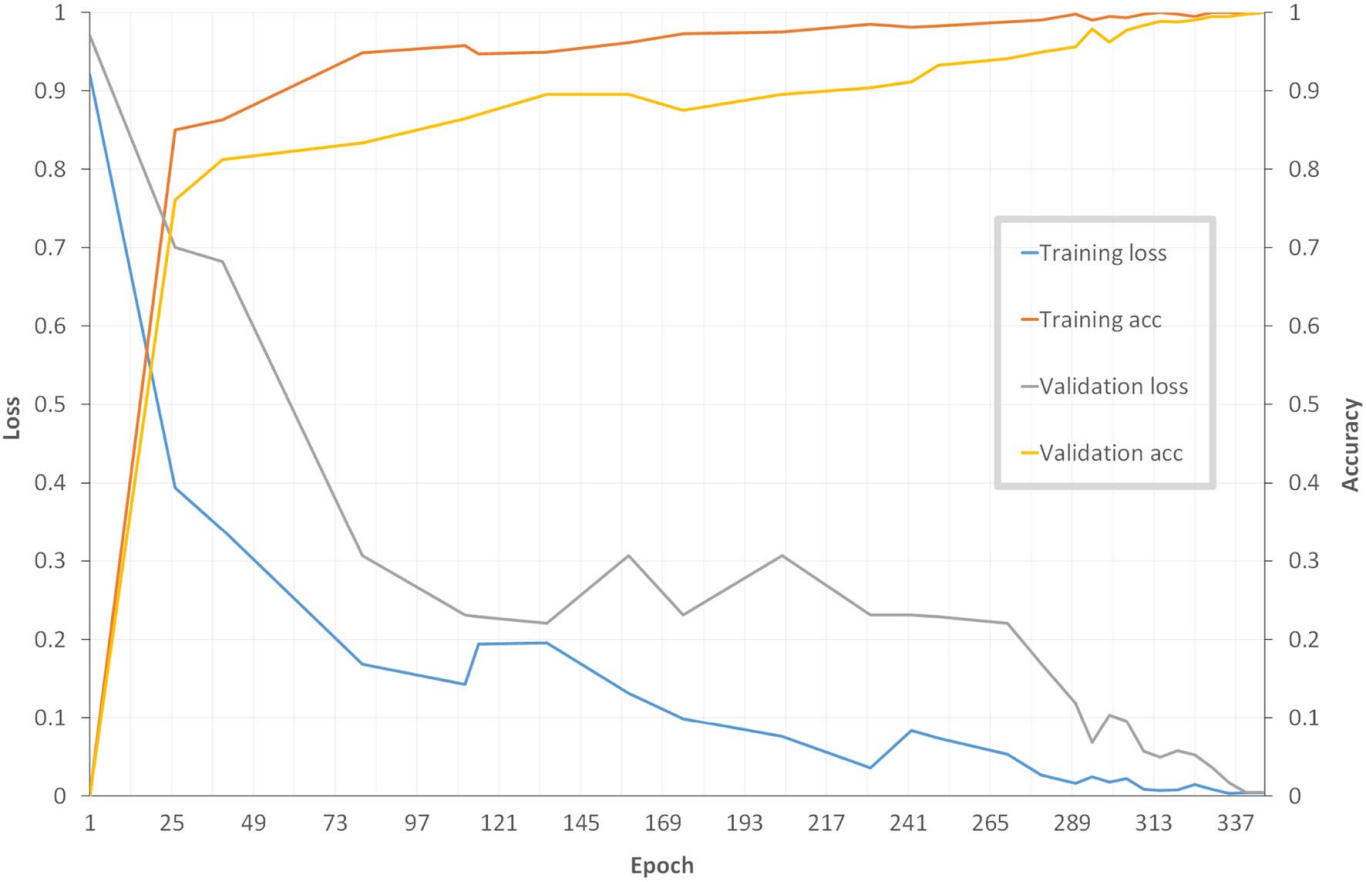
Plots of accuracy and loss for the classification of the training and validation images, as obtained by TRDCNN.

Multiple tests were conducted to confirm the suggested network’s performance in achieving the tumor-detection goal. Table 4 shows a comparison with various methods reported in the literature. The empirical results demonstrate that the suggested TRDCNN structure outperforms existing techniques in terms of accuracy, sensitivity, and specificity. Without taking network training time into account, the average testing duration per picture was 0.28 s. Figure 7 shows the improvement TRDCNN achieves over the training and validation images.

**Table 4:** Comparison of the proposed approach against some methods in the literature.

Methods	Evaluation metrics (%)			
	SV	SP	AC	IoU
Pan et al.^74^	73.33	73.33	73.33	57.89
Abd-Ellah et al.^3^	83.43	25.00	66.96	51.28
Ye et al.^75^	88.90	57.00	82.10	69.44
Ge et al.^76^	–	–	89.47	80.35
Heba et al.^29^	97.00	97.00	96.97	93.26
Sultan et al.^77^	94.40	95.10	95.81	91.56
Anaraki et al.^78^	98.30	95.70	96.50	90.40
Abd-Ellah et al.^79^	97.00	98.00	97.44	95.40
Tazin et al.^30^	94.04	90.00	92.00	86.81
Mudda et al.^27^	52.90	93.00	94.00	88.23
Alsaif et al.^31^	93.00	100.00	96.00	92.90
Asiri et al.^28^	90.70	90.70	90.70	86.39
Proposed TRDCNN	98.66	99.00	98.88	97.81

### Analysis of tumor-segmentation accuracy

**Figure 8:**
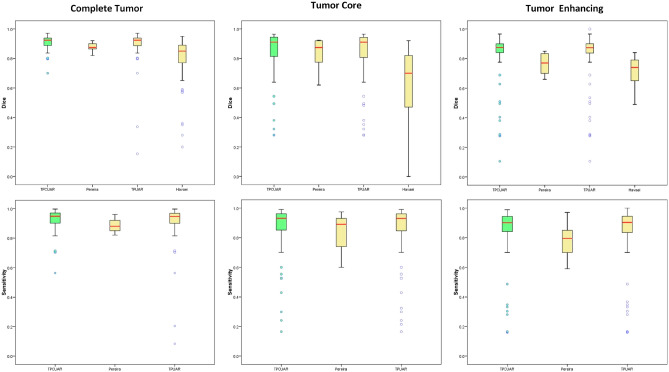
Dice scores and sensitivity; the median is shown in red, and outliers are in blue.

**Figure 9:**
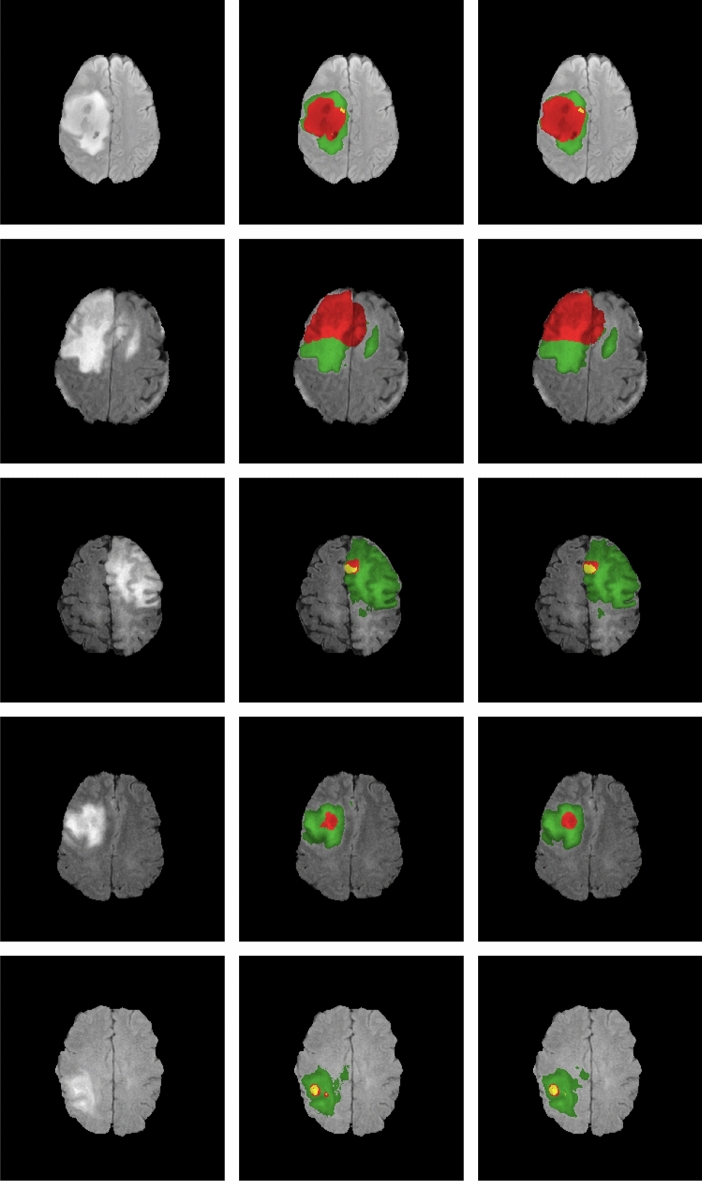
Visual results from TPCUAR-Net from the axial view. From first to third, the columns show the FLAIR modality of HG and LG tumors, the ground truth, and the predicted image.

Table 5 presents the performance of TPCUAR-Net in comparison with different investigations found in the literature that used the U-net network. Reproduction tests have been directed to demonstrate the execution of the presented system in fulling the segmentation assignment. A comparison with other investigations in the literature is given in Table 6, and Fig. 9 presents the segmentation findings. Without taking network training time into account, the average testing duration per picture was 0.08 s. Figure 8 presents boxplots for the test dataset. In this figure, our proposed method ranks first among competing results for complete, core, and enhancing tumors, with fewer outliers than the other techniques.

**Table 5:** Comparison of the proposed TPCUAR-Net against some methods based on U-Net in terms of Dice score.

Methods	Dice score		
	Complete	Core	Enhancing
3D U-Net^86^	0.86	0.76	0.73
3D U-Net+TTA^86^	0.87	0.78	0.75
U-Net+ZP^72^	0.86	0.86	0.65
U-Net+ET^87^	0.85	0.81	0.72
TPUAR-Net^2^	0.89	0.82	0.79
Proposed TPCUAR-Net	0.91	0.83	0.80

**Table 6:** Comparison of the proposed TPCUAR-Net against some state-of-the-art methods.

Methods	Dice score			Sensitivity			Specificity			Hausdorff		
	Comp	Core	Enha	Comp	Core	Enha	Comp	Core	Enha	Comp	Core	Enha
Zhao et al.^71^	0.87	0.83	0.76	0.83	0.81	0.77	0.92	0.87	0.77	–	–	–
Pereira et al.^62^	0.85	0.76	0.74	0.92	0.79	0.78	0.80	0.78	0.74	–	–	–
Pereira et al.^61^	0.84	0.72	0.62	0.89	0.83	0.81	0.88	0.87	0.74	–	–	–
Mohamad et al.^4^	0.88	0.79	0.73	0.87	0.79	0.80	0.89	0.79	0.68	–	–	–
Kamnitsas et al.^64^	0.85	0.67	0.63	0.85	0.84	0.63	0.87	0.61	0.66	39.61	–	–
Zhao et al.^63^	0.88	0.84	0.77	0.90	0.87	0.76	0.86	0.82	0.80	–	–	–
Abd-Ellah et al.^2^	0.89	0.82	0.79	0.89	0.84	0.81	0.99	0.99	0.99	5.47	7.48	9.94
Zhang et al.^67^	0.90	0.82	0.75	0.90	0.79	0.75	–	–	–	5.155	6.999	3.170
Remya et al.^68^	0.72	0.70	0.69	–	–	–	–	–	–	–	–	–
Wang et al.^66^	0.89	0.84	0.81	0.91	0.85	0.84	0.99	1	1	6.24	19.54	17.79
Myronenko^69^	0.88	0.81	0.76	–	–	–	–	–	–	5.90	4.80	3.77
TPCUAR-Net	0.91	0.83	0.80	0.92	0.85	0.81	0.99	0.99	0.99	3.31	1.41	2.23

### Discussion

The detection and classification of brain tumors are important steps that rely on the expertise and knowledge of a physician, and an intelligent method for detecting and classifying brain tumors is vital for assisting clinicians. Gliomas, with their uneven shapes and uncertain boundaries, are the most difficult tumors to diagnose. Image segmentation presents substantial issues in terms of categorization, image processing, object detection, and explanation. For example, whenever an image-classification model is developed, it must be able to work with great precision even when subjected to occlusion, lighting variations, viewing angles, and other factors.

In this study, we designed a deep CNN diagnosis model based on parallel paths. This can provide various benefits over and above those of a standard CNN, including increased model capacity and feature diversity, ensemble learning, multi-resolution analysis, efficient information flow, and regularization capabilities. These advantages can boost the network’s performance, its capacity to learn complex patterns, and its generalization ability. We then incorporated residual blocks into our model to achieve improved performance, providing benefits such as increased model capacity, improved gradient flow, enhanced feature reuse, regularization for reduced overfitting, efficient training, and flexibility in network design. The addition of residual blocks can help to train deep models faster and more efficiently. Skip connections are used to provide faster convergence by giving direct access to lower-level capabilities. This minimizes the number of weight updates necessary for information propagation, resulting in faster training overall. Efficiency in training is especially important when dealing with huge datasets and computationally expensive models. The proposed detection and classification model was found to achieve an accuracy of 98.88%, which is greater than the accuracy of other models reported in the literature. In the segmentation, our architecture achieved a Dice score of 0.91, which is greater than those of other comparable models reported in the literature. Therefore, increasing the depth of U-Net by using cascaded and parallel paths with the application of some useful preprocessing techniques for can improve the segmentation performance.

The processing time is another important factor for evaluating the proposed model. The training time was not considered, because the parameters were kept unchanged after training. The technique used for measuring processing time involved transmitting all of the images into the proposed system, recording the associated calculation time for each stage for each individual image, and computing the average value to reflect the times used by different stages. The average testing duration per image was 0.41 s.

Our innovative complete CADx approach has the potential to play an important role in the early detection and diagnosis of brain tumors; it can be applied as a useful tool in hospital emergency units during the examination of patients with MRI scans because of the greater possible speed of diagnosis. Our method allows doctors to analyze MRI images before processing to determine whether they are normal or contain an HGG or LGG. Once an image is recognized as abnormal, clinicians can detect and segment the brain tumor to determine its size.

## Conclusions and future work

Herein, we have described a deep-learning-based technique for detecting, classifying, and segmenting glioblastoma brain tumors using MRI images. The primary goal of this work was to merge the detection, classification, and segmentation processes into a single fully automated system. The first phase includes deep CNN architecture for brain-tumor identification and classification from MRI scans, which classifies the pictures as normal, HGG, or LGG using the TRDCNN architecture. This was assessed using the BraTS 2017 database, with 1350 and 450 images used for training and testing, respectively. The TRDCNN architecture was found to produce encouraging results in terms of accuracy, sensitivity, and specificity, with values of 98.88%, 98.66%, and 99.60%, respectively.

For the second phase, a deep-CNN-based automatic brain-tumor segmentation approach from MRI images was described. Various structures with varying depths were studied and investigated. The second phase was assessed using a database derived from the BraTS 2017 dataset, with 62,000 and 43,400 images used for training and testing, respectively. The TPCUAR-Net was found to produce the best results for the total, core, and enhancing tumor areas, with a maximum Dice score of 0.91 and a testing duration of 0.45 s per image. The suggested method’s superiority stems from various advantages, including its ability to evaluate both local and global aspects to learn both high-level and low-level information at the same time. The use of a fully connected layer, residual blocks, and skip connections could help to solve the vanishing-gradient problem while also speeding up both training and testing. A cascaded network can efficiently train a CNN when the label distribution is imbalanced. In the two parallel networks, the proposed technique integrates both global and local features.

One factor contributing to this is the lack of defined procedures for evaluating CADx systems in a real setting. The image formats used to train the models were those of the AI research area (PNG) rather than those of the radiology field (DICOM, NIfTI), which is significant. Furthermore, the analysis requires authors with clinical backgrounds. As such, the engagement of doctors in the process may benefit the study project’s relevance and the acceptance of its findings. Our planned future work involves implementing complete CADx systems within clinical practice. The existing design will be improved in the future, with a possible expansion to a 3D CNN architecture, enabling 3D brain-tumor diagnosis from MRI and other scans.

### Supplementary Information

Supplementary Information.

## Data Availability

Data are available from the corresponding author upon request. The BraTS 2017 dataset is publicly available at https://www.med.upenn.edu/sbia/brats2017/data.html or https://www.kaggle.com/datasets/xxc025/unet-datasets?select=BRATS2017.zip.
