# Supplementary figures and images for: Automatic brain-tumor diagnosis using cascaded deep convolutional neural networks with symmetric U-Net and asymmetric residual-blocks
Source: Sci Rep. 2024 Apr 25;14:9501. doi: 10.1038/s41598-024-59566-7 (PMC11045751; doi:10.1038/s41598-024-59566-7)

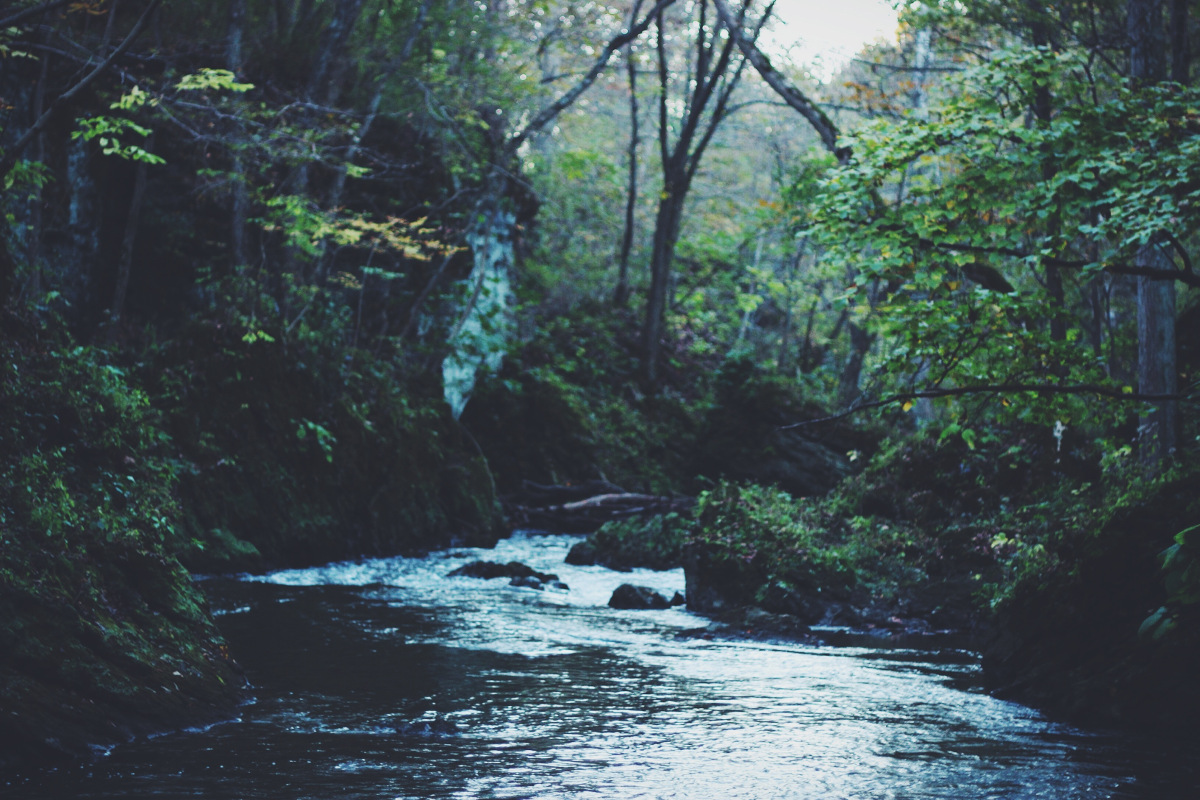

Supplement: Supplementary file 1 — Supplementary Information. [file 41598_2024_59566_MOESM1_ESM.zip › stream.jpg]

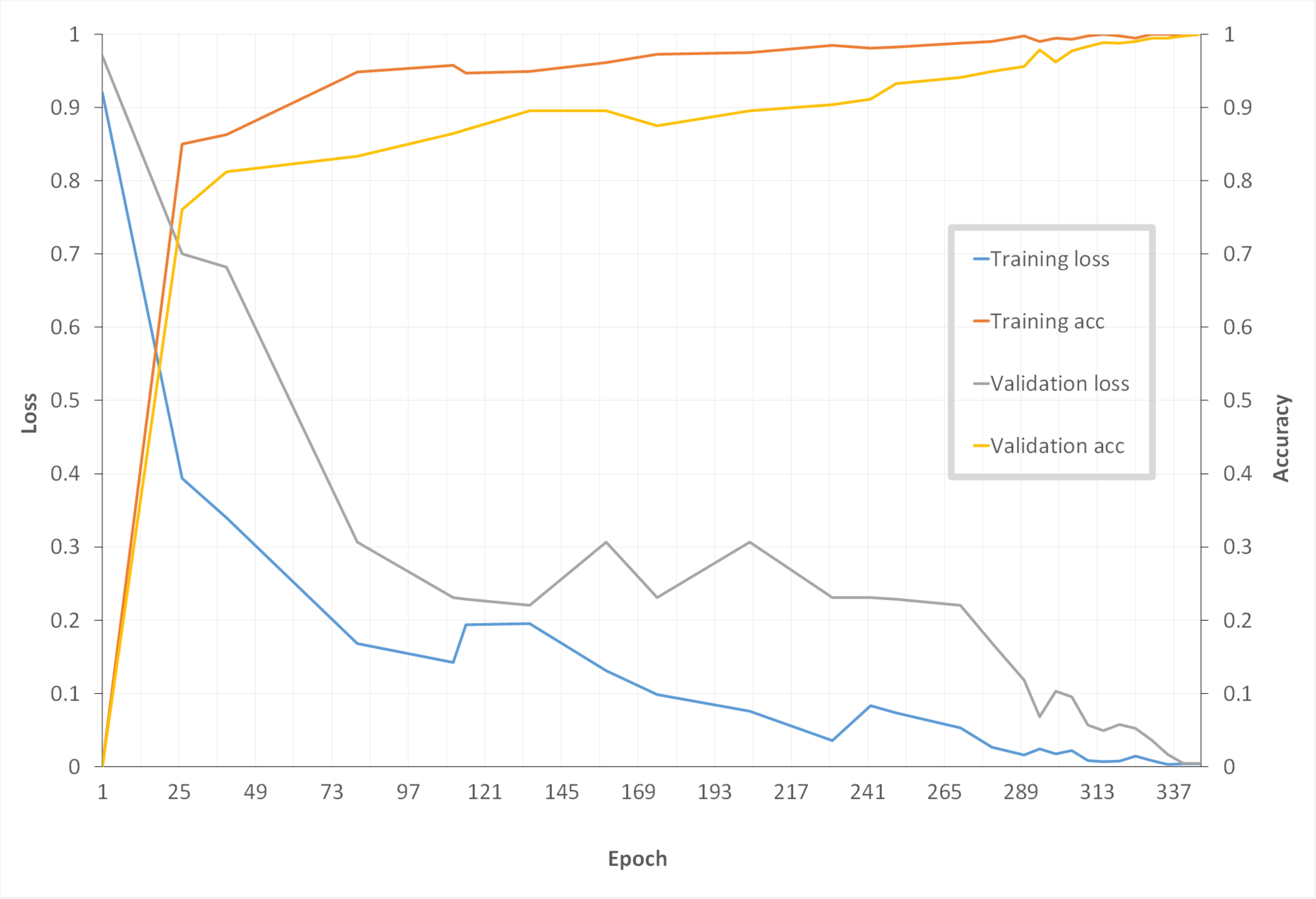

Supplement: Supplementary file 1 — Supplementary Information. [file 41598_2024_59566_MOESM1_ESM.zip › Figures/Figure-06.jpg]

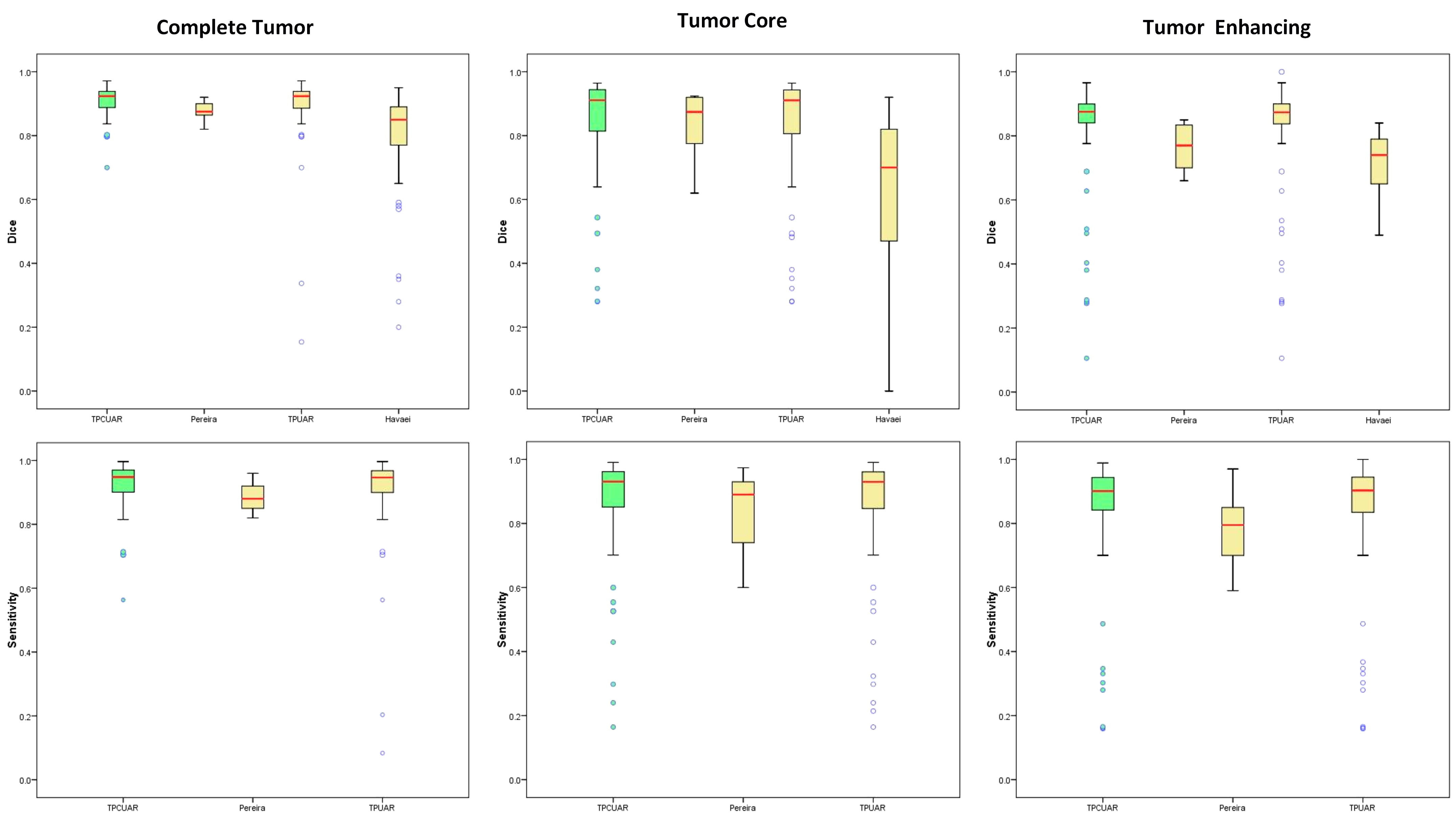

Supplement: Supplementary file 1 — Supplementary Information. [file 41598_2024_59566_MOESM1_ESM.zip › Figures/Figure-007.jpg]

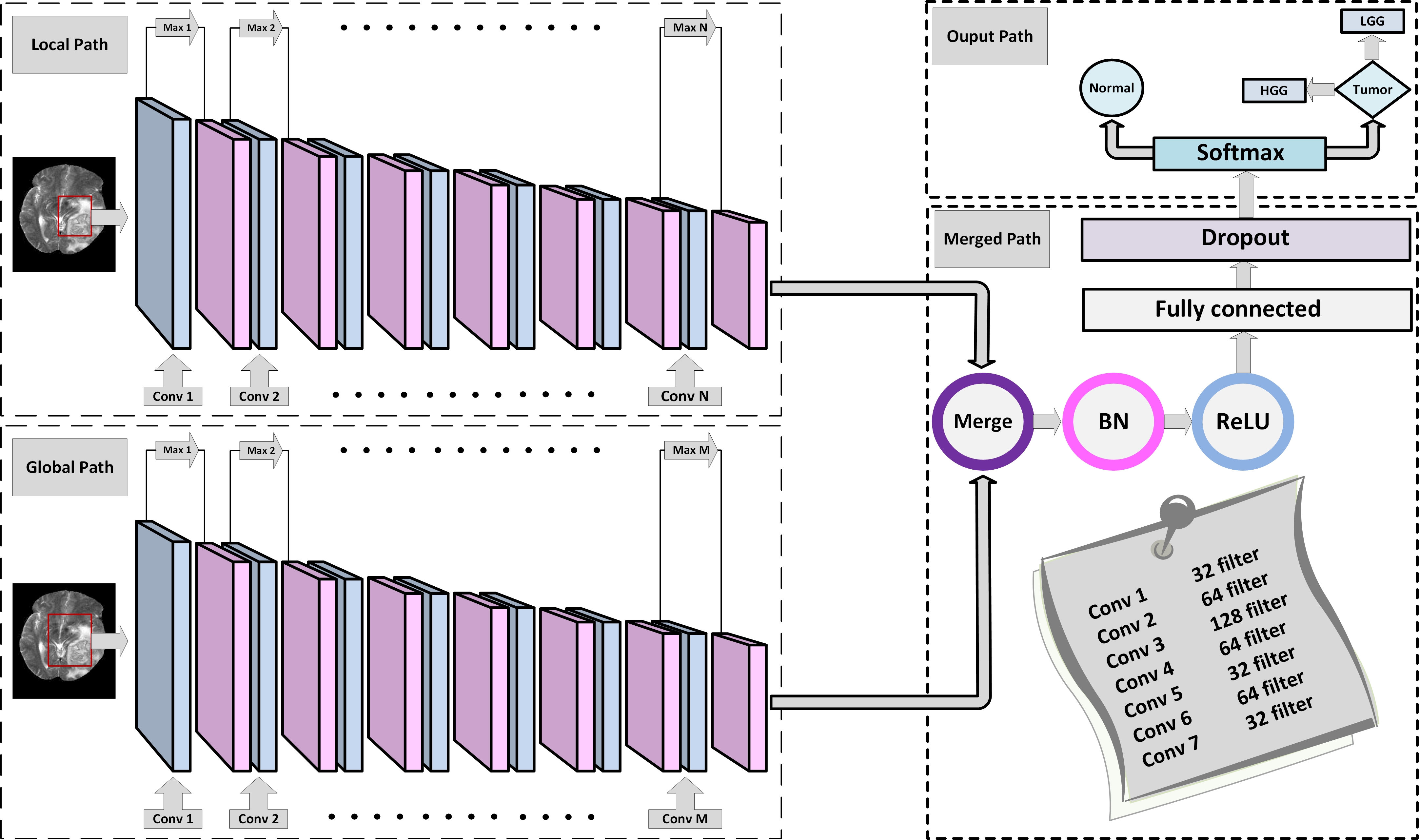

Supplement: Supplementary file 1 — Supplementary Information. [file 41598_2024_59566_MOESM1_ESM.zip › Figures/Figure-011.jpg]

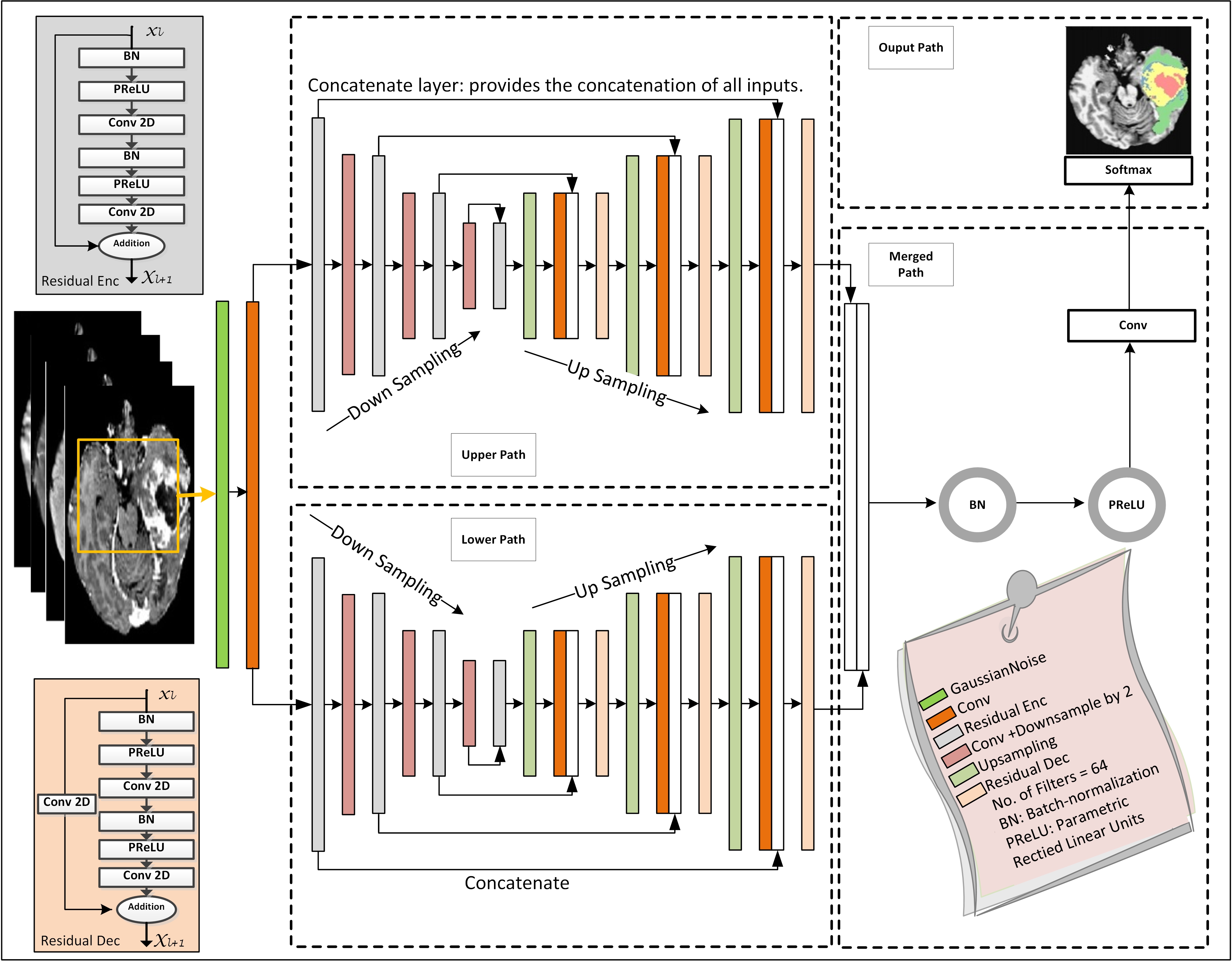

Supplement: Supplementary file 1 — Supplementary Information. [file 41598_2024_59566_MOESM1_ESM.zip › Figures/Figure-0021.jpg]

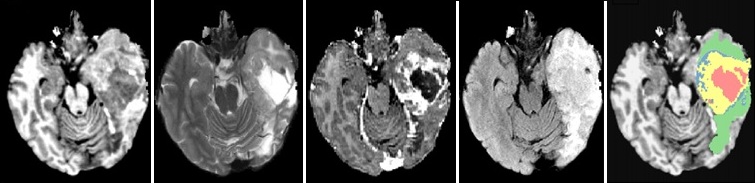

Supplement: Supplementary file 1 — Supplementary Information. [file 41598_2024_59566_MOESM1_ESM.zip › Figures/Figure-02.jpg]

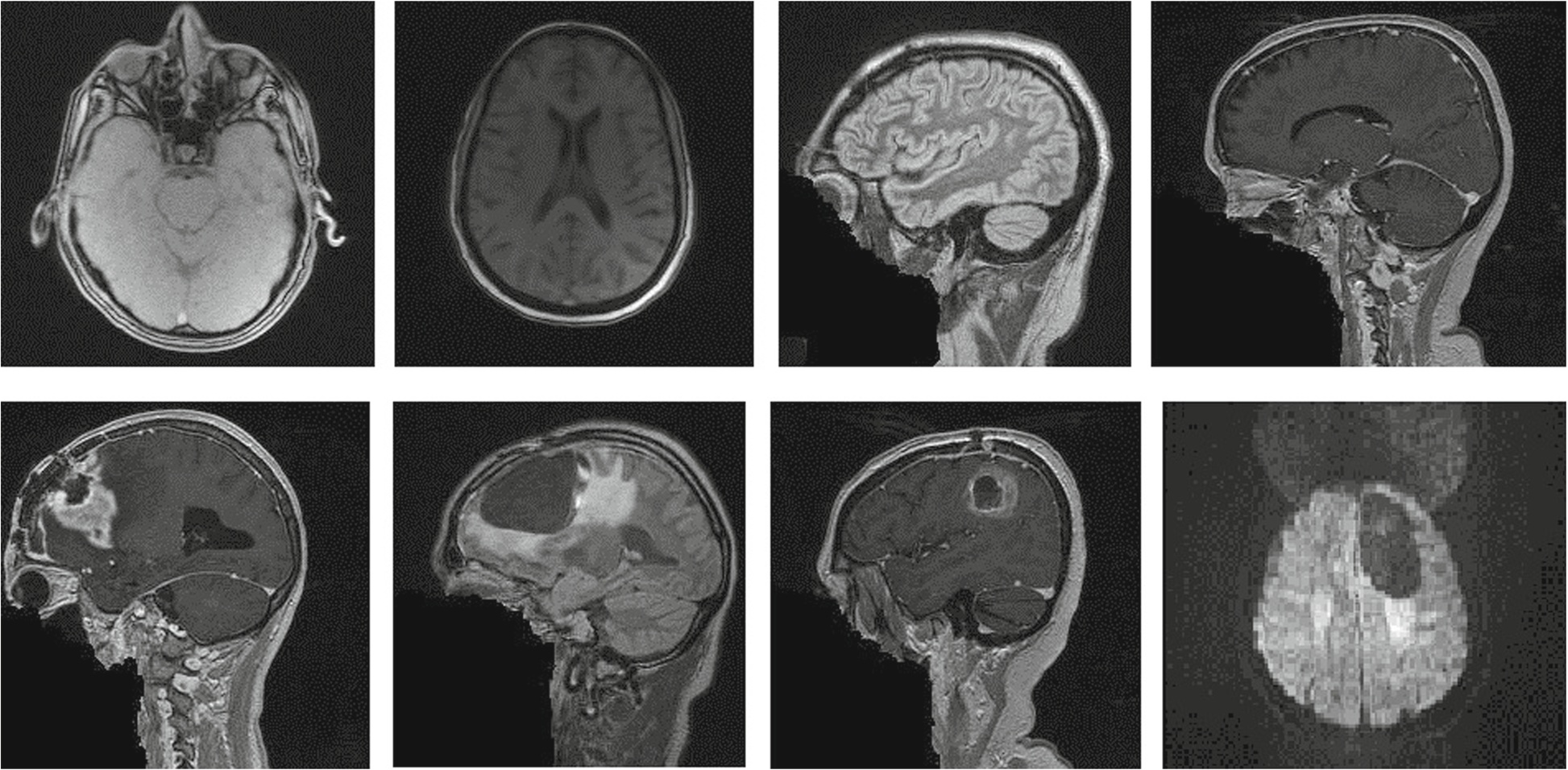

Supplement: Supplementary file 1 — Supplementary Information. [file 41598_2024_59566_MOESM1_ESM.zip › Figures/Figure-04.jpg]

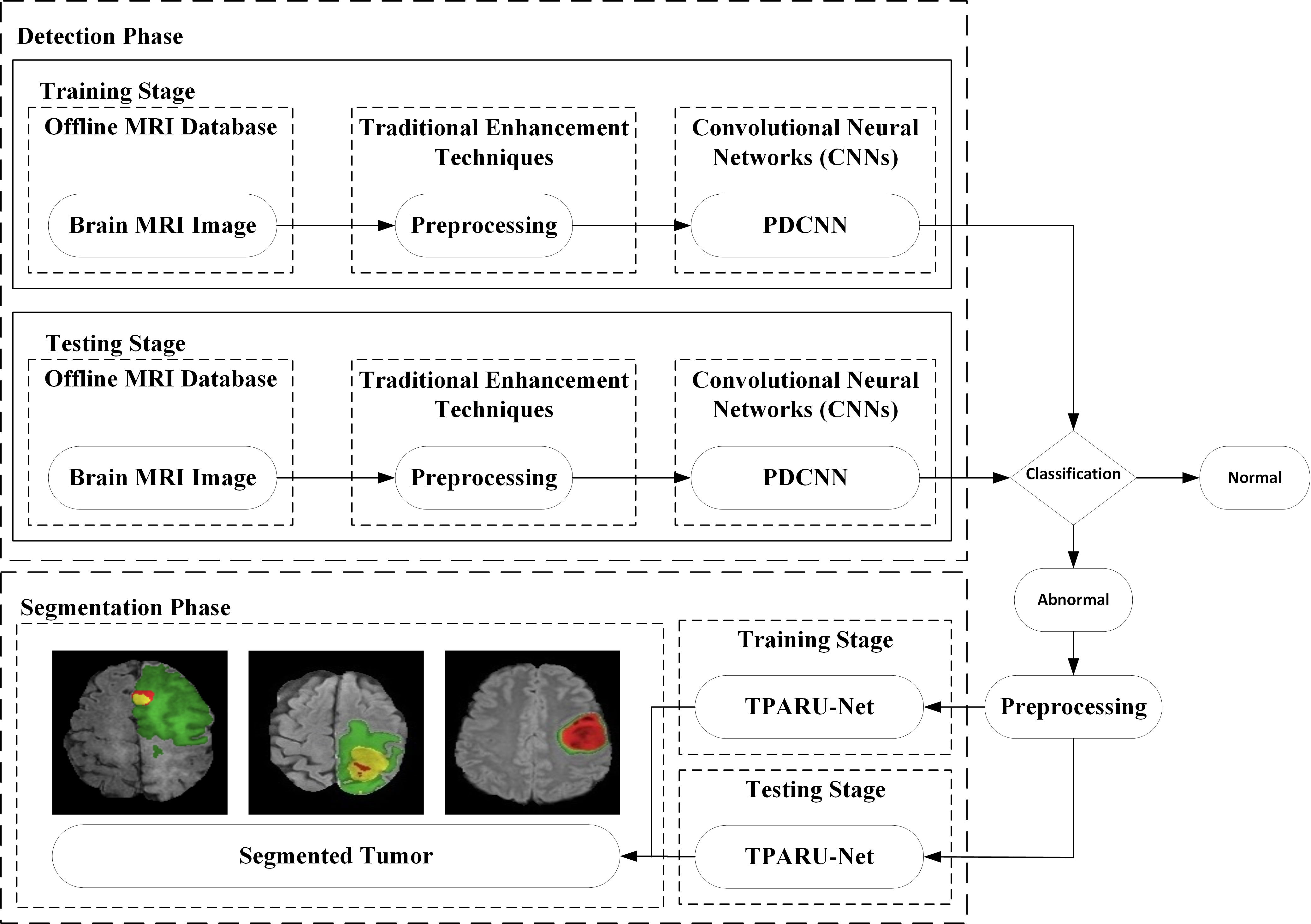

Supplement: Supplementary file 1 — Supplementary Information. [file 41598_2024_59566_MOESM1_ESM.zip › Figures/Figure-05.jpg]

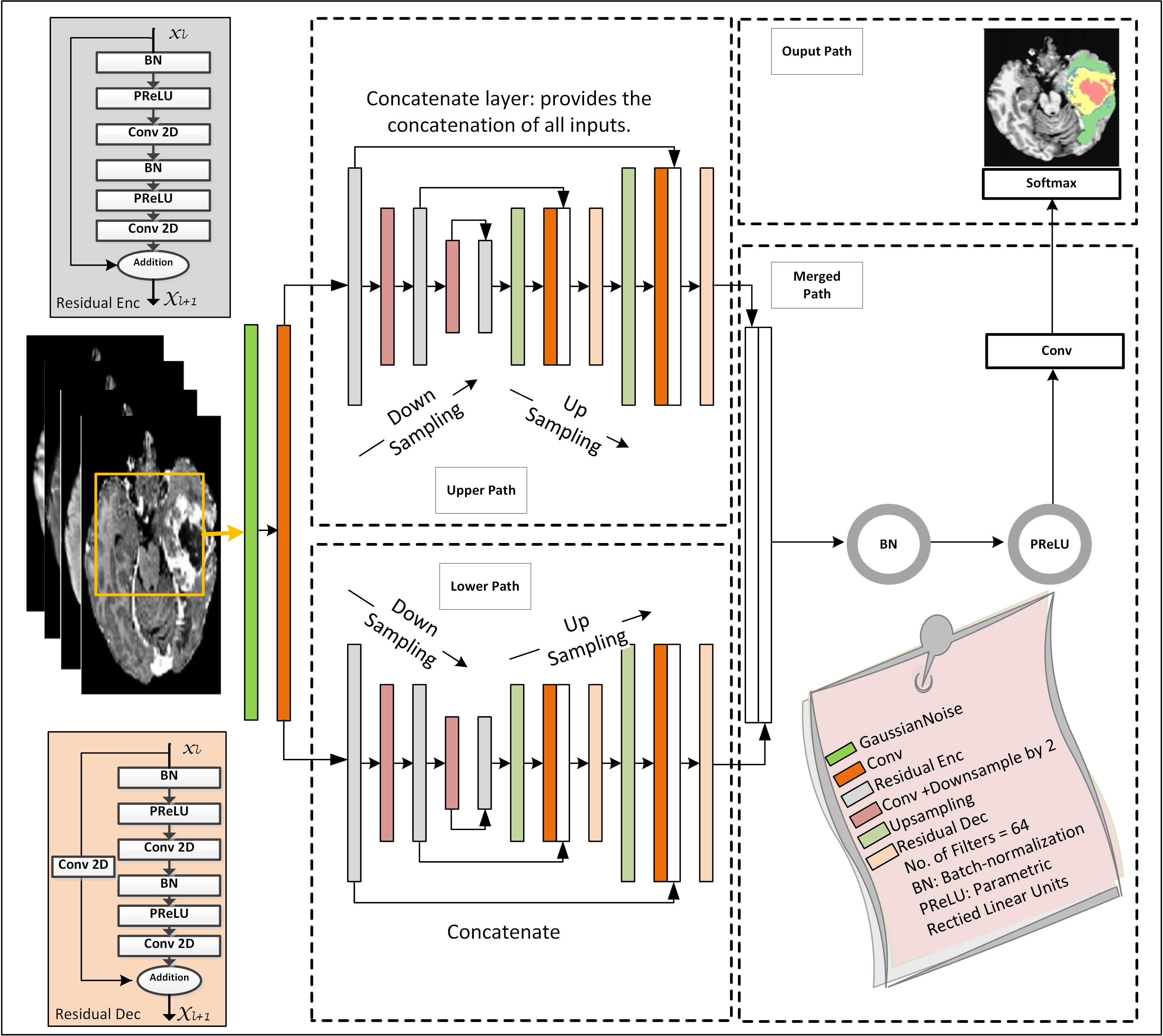

Supplement: Supplementary file 1 — Supplementary Information. [file 41598_2024_59566_MOESM1_ESM.zip › Figures/Figure-0022.jpg]

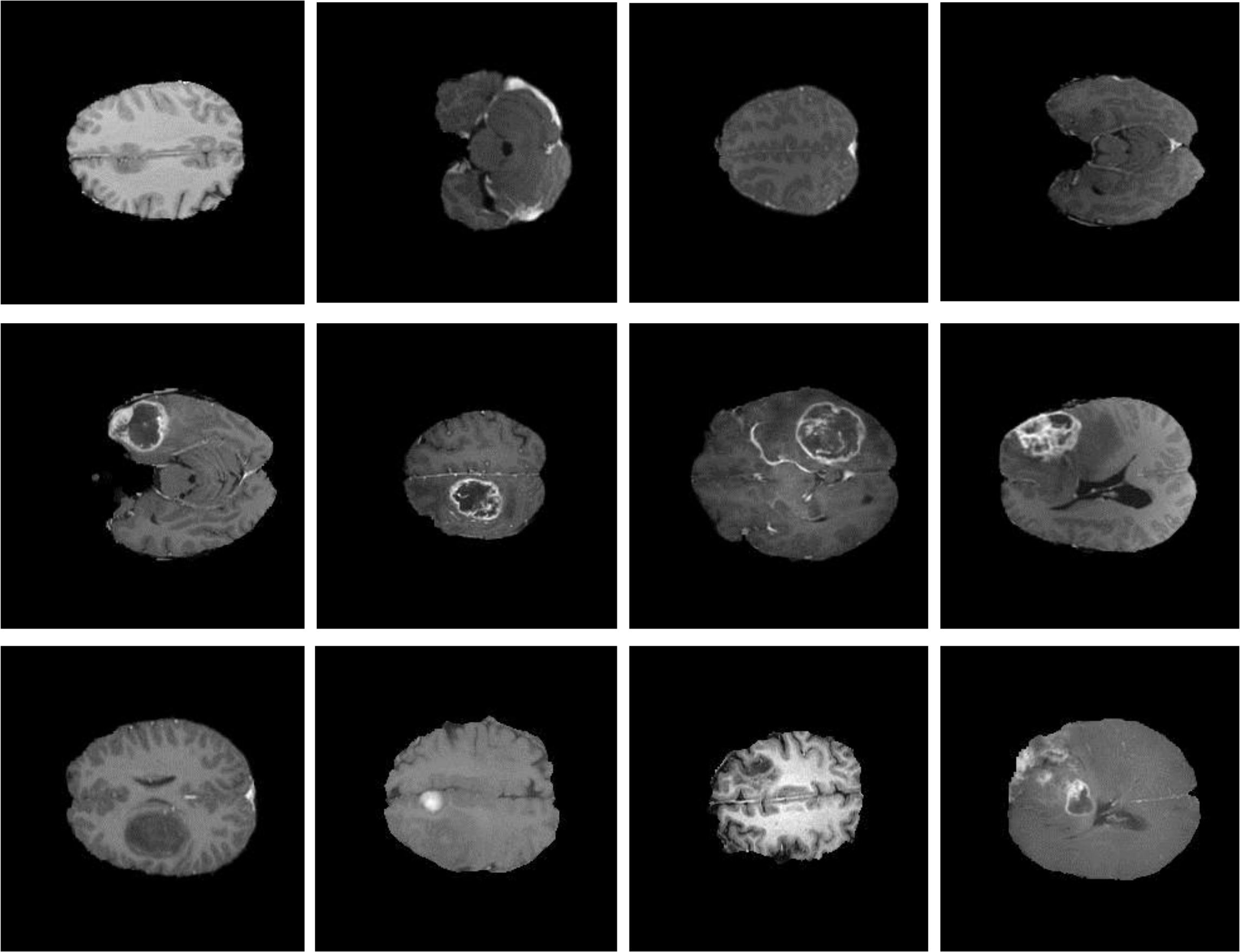

Supplement: Supplementary file 1 — Supplementary Information. [file 41598_2024_59566_MOESM1_ESM.zip › Figures/Figure-041.jpg]

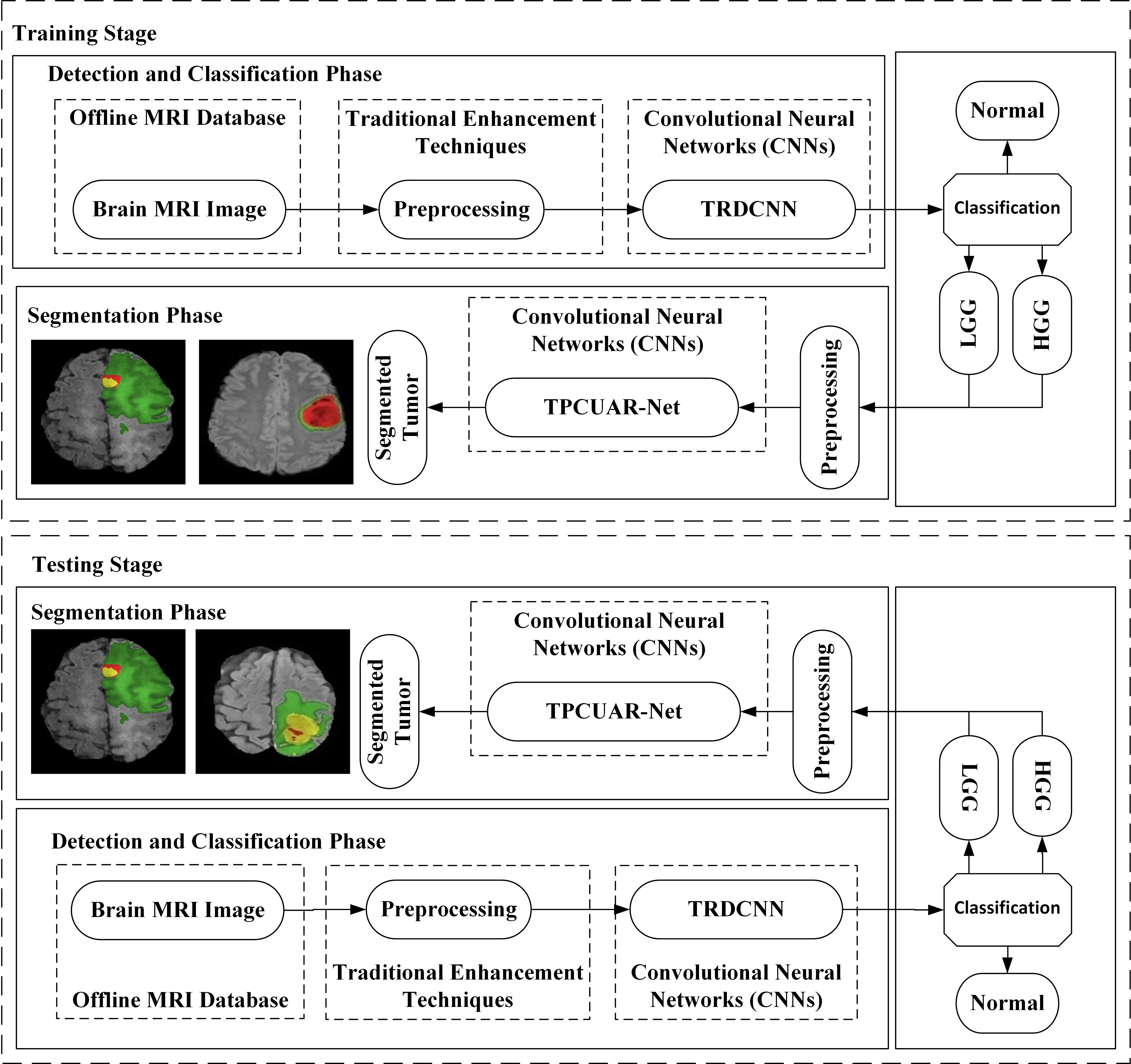

Supplement: Supplementary file 1 — Supplementary Information. [file 41598_2024_59566_MOESM1_ESM.zip › Figures/Figure-051.jpg]

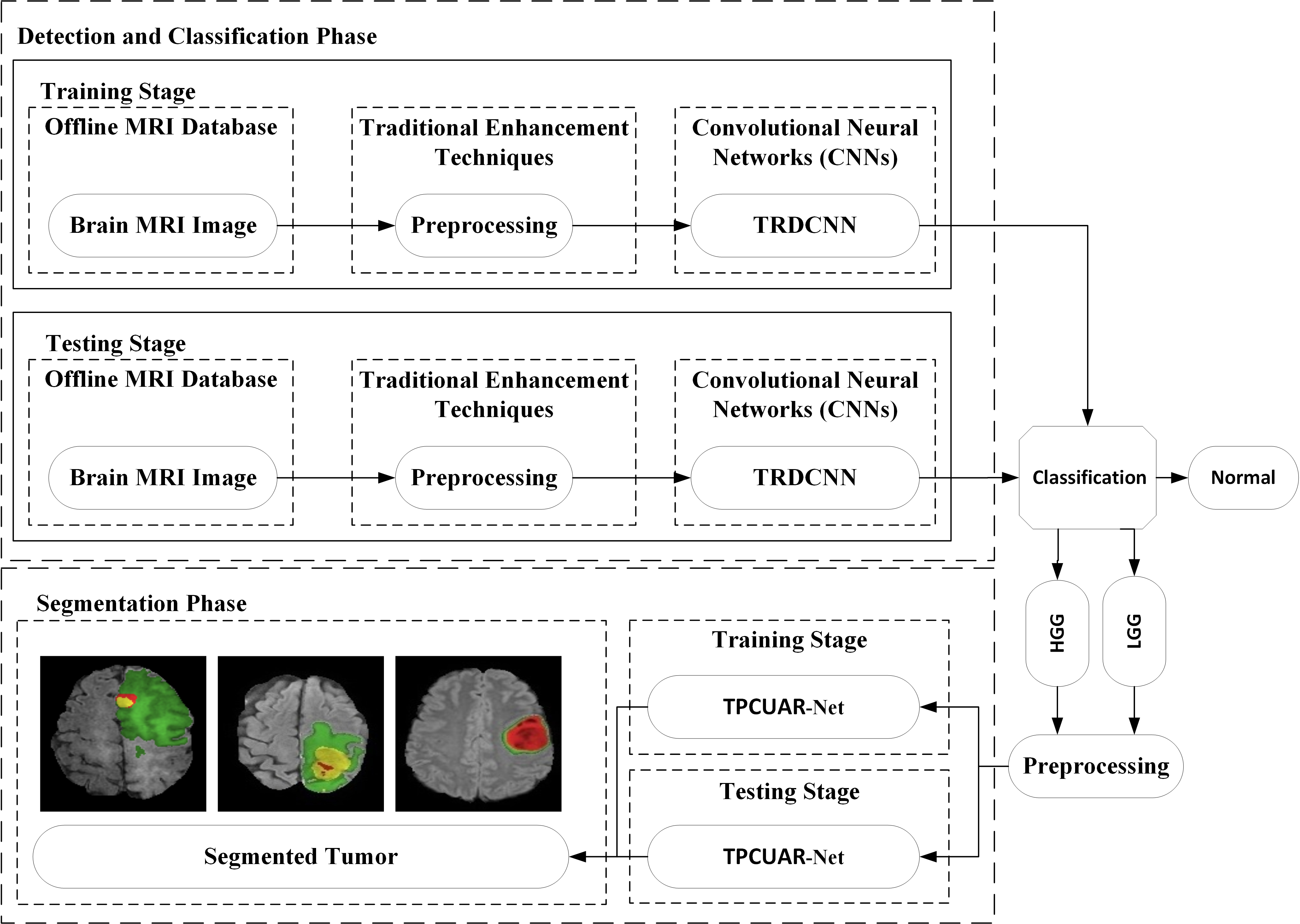

Supplement: Supplementary file 1 — Supplementary Information. [file 41598_2024_59566_MOESM1_ESM.zip › Figures/Figure-0511.jpg]

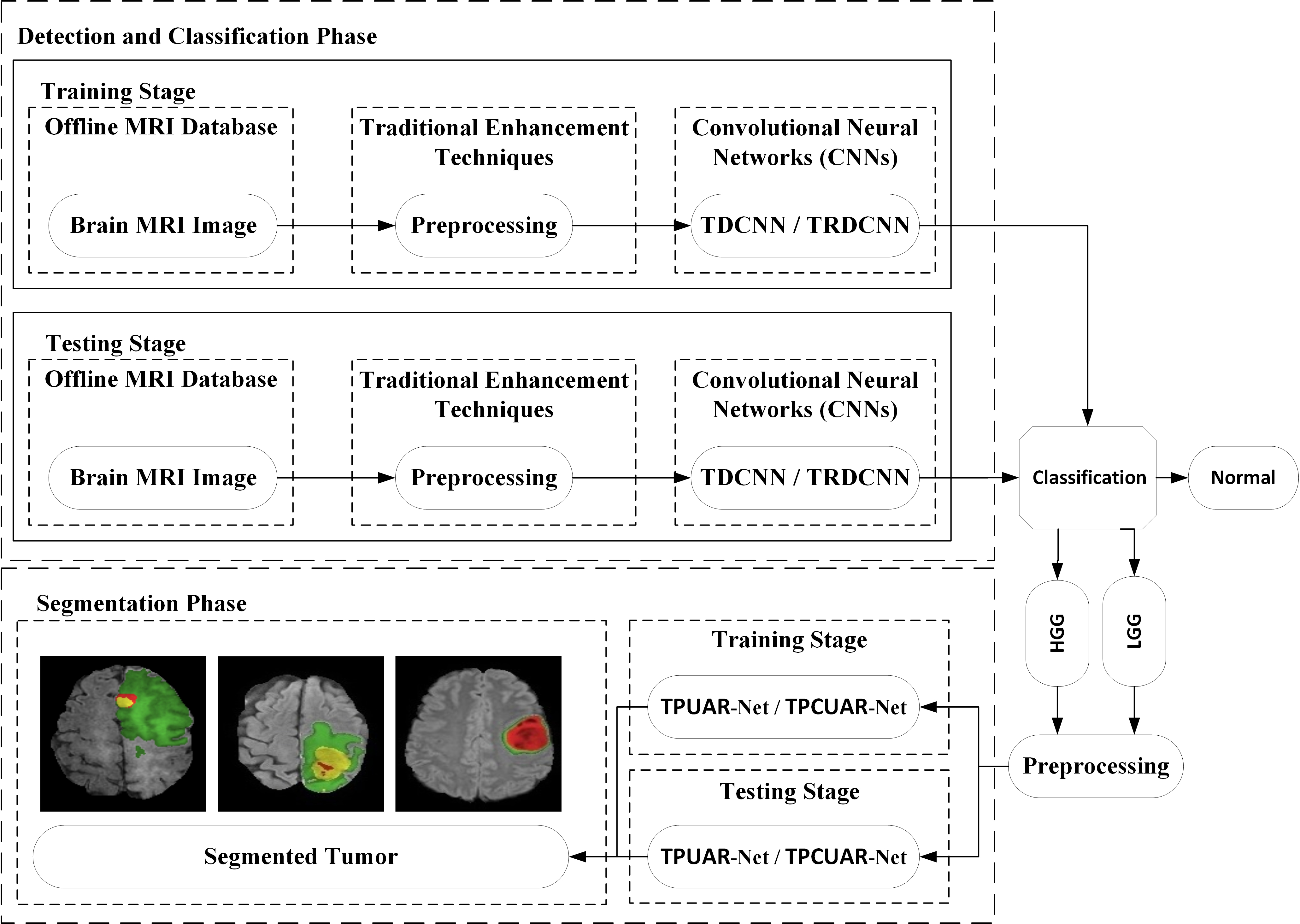

Supplement: Supplementary file 1 — Supplementary Information. [file 41598_2024_59566_MOESM1_ESM.zip › Figures/Figure-0513.jpg]

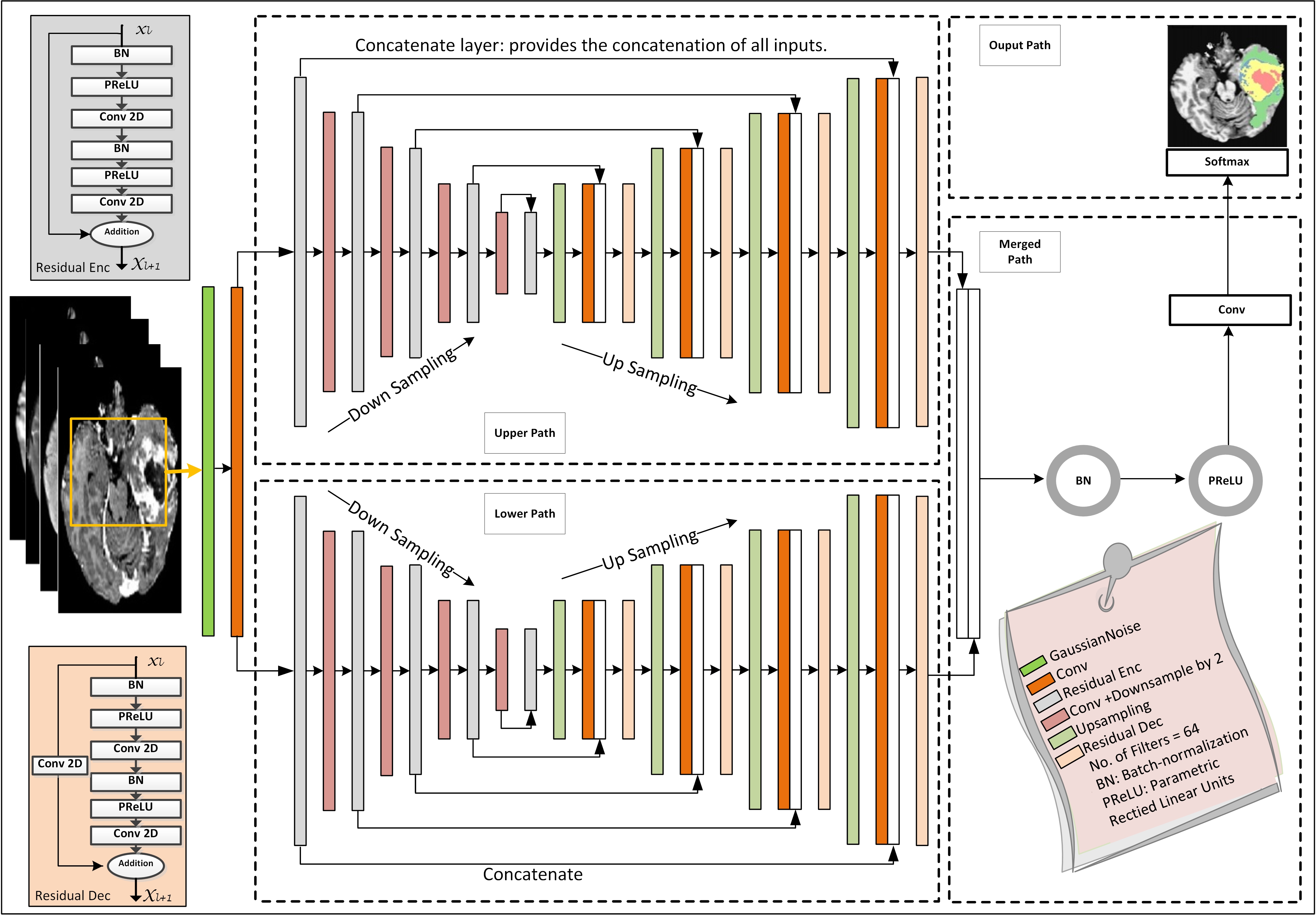

Supplement: Supplementary file 1 — Supplementary Information. [file 41598_2024_59566_MOESM1_ESM.zip › Figures/PRDCNN.jpg]

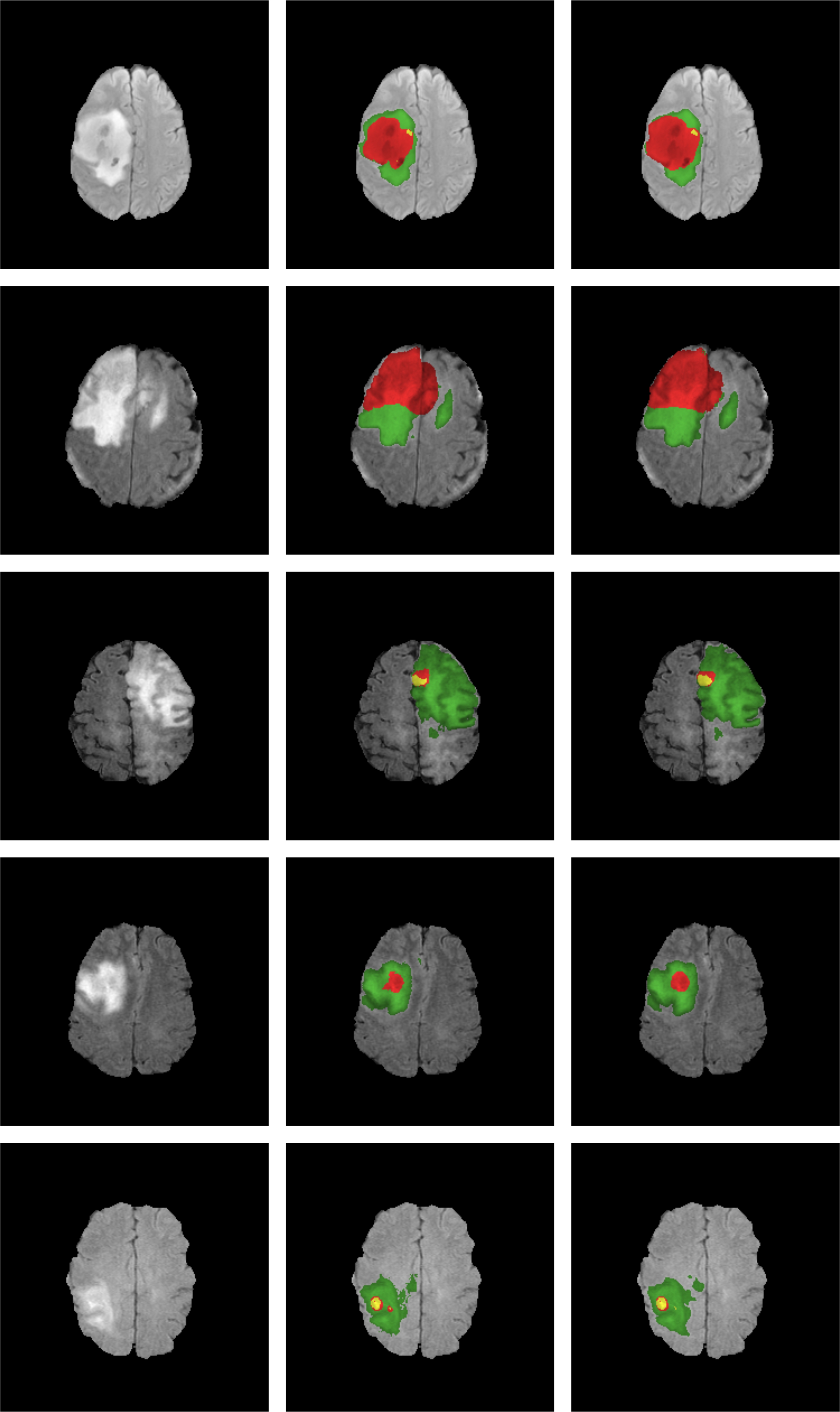

Supplement: Supplementary file 1 — Supplementary Information. [file 41598_2024_59566_MOESM1_ESM.zip › Figures/result.jpg]

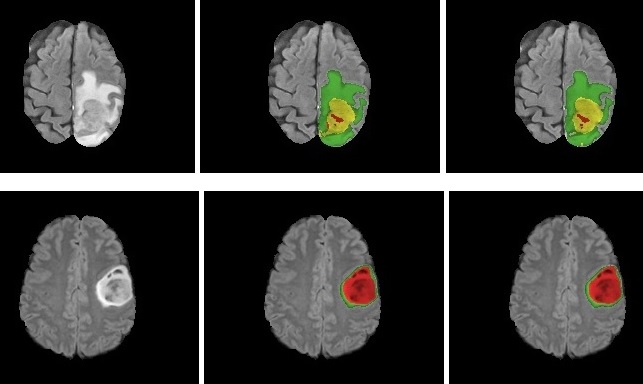

Supplement: Supplementary file 1 — Supplementary Information. [file 41598_2024_59566_MOESM1_ESM.zip › Figures/Results.jpg]

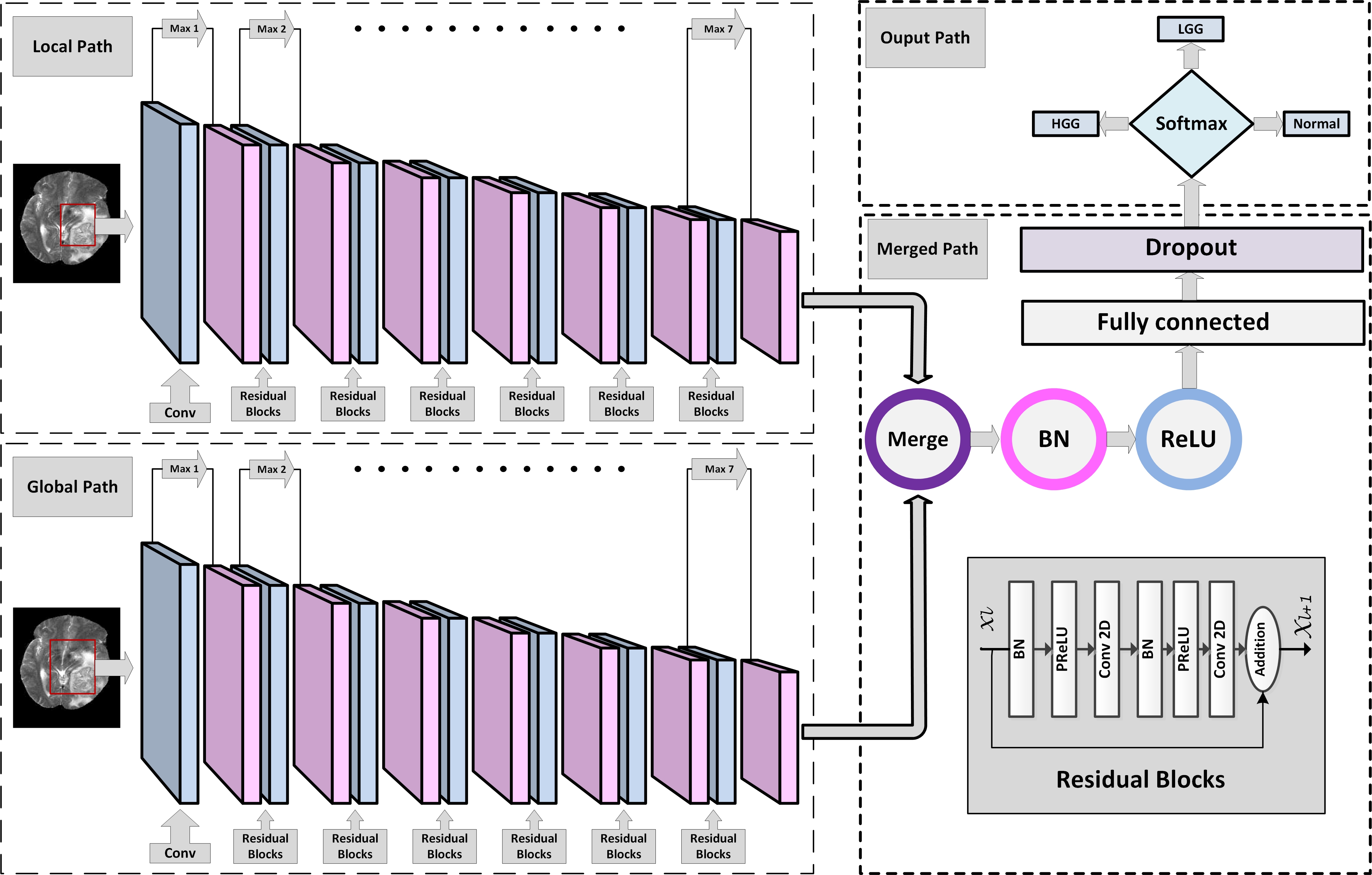

Supplement: Supplementary file 1 — Supplementary Information. [file 41598_2024_59566_MOESM1_ESM.zip › Figures/Figure-001.jpg]

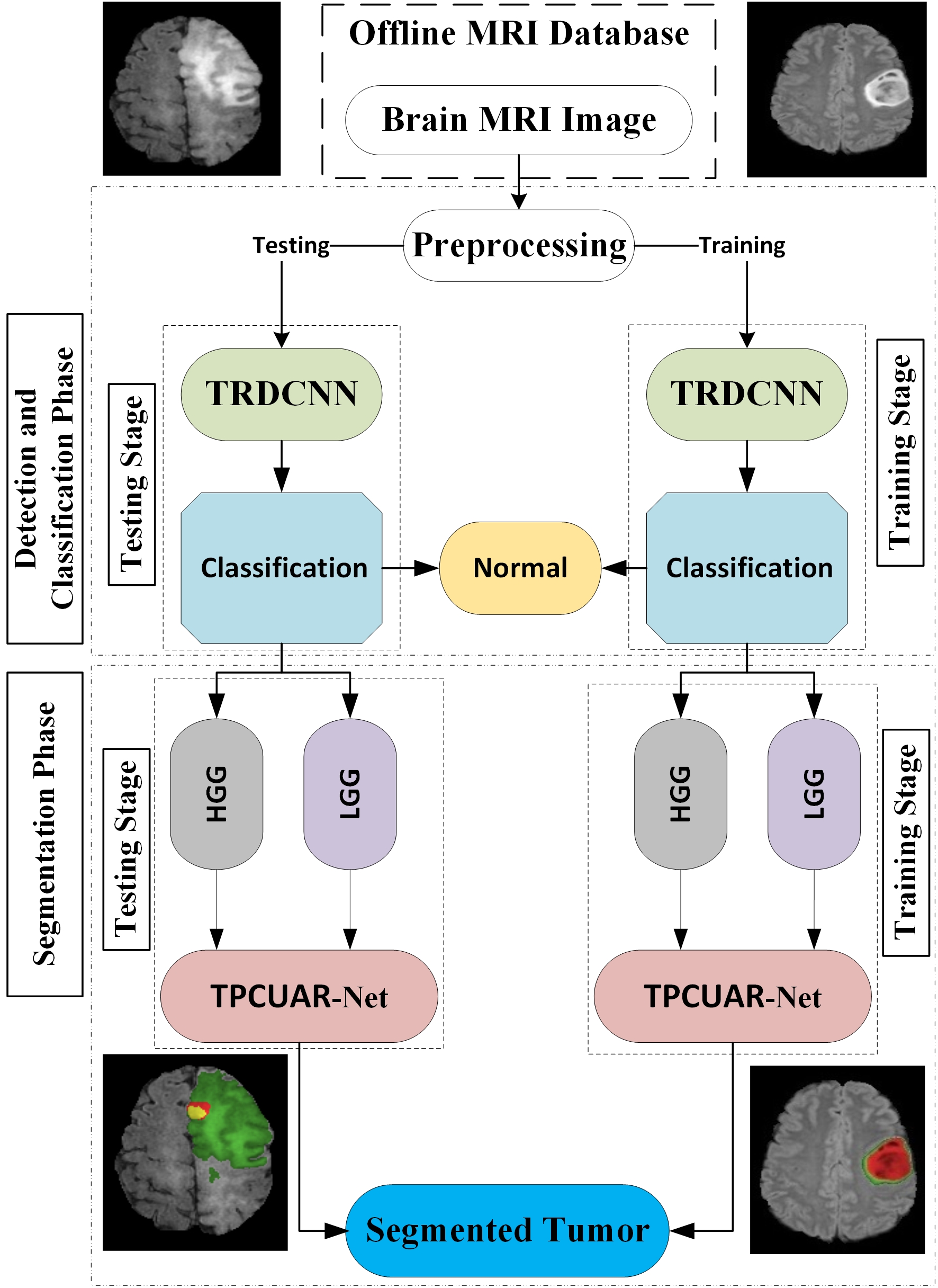

Supplement: Supplementary file 1 — Supplementary Information. [file 41598_2024_59566_MOESM1_ESM.zip › Figures/Figure-055.jpg]

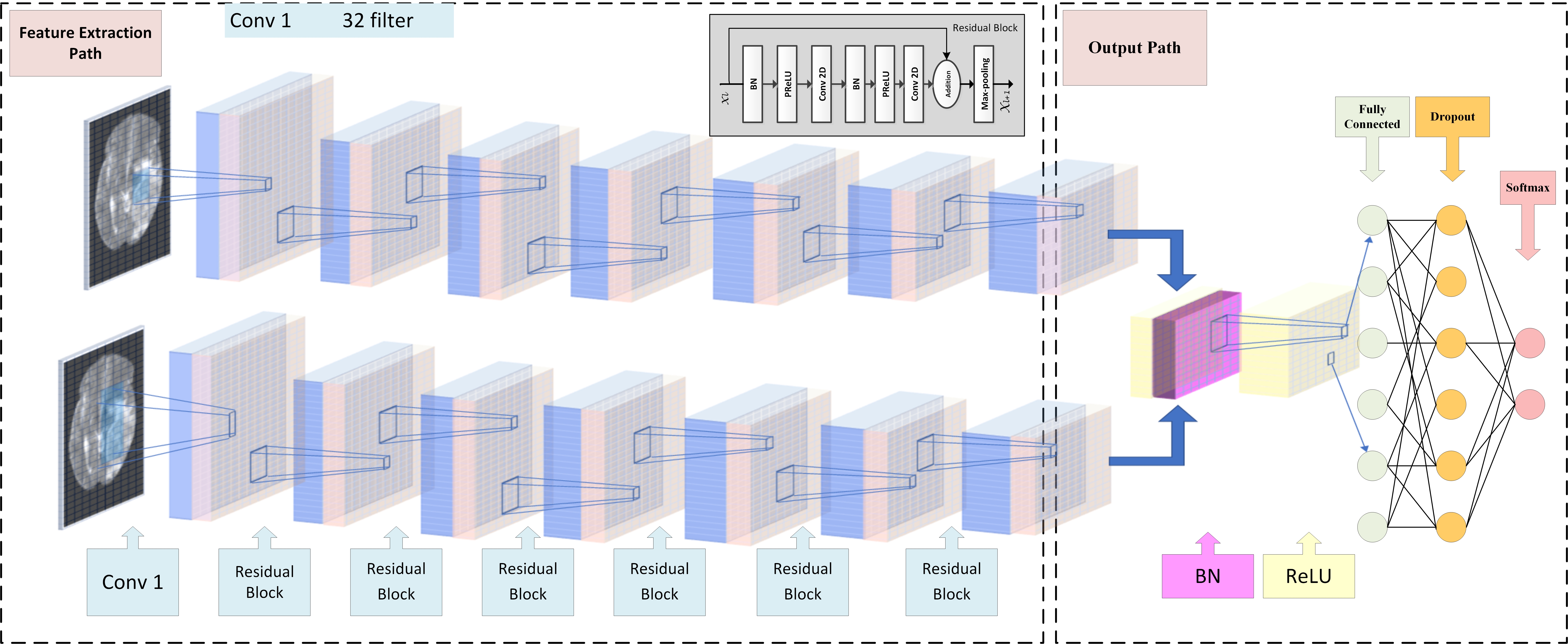

Supplement: Supplementary file 1 — Supplementary Information. [file 41598_2024_59566_MOESM1_ESM.zip › Figures/Figure-001a.jpg]

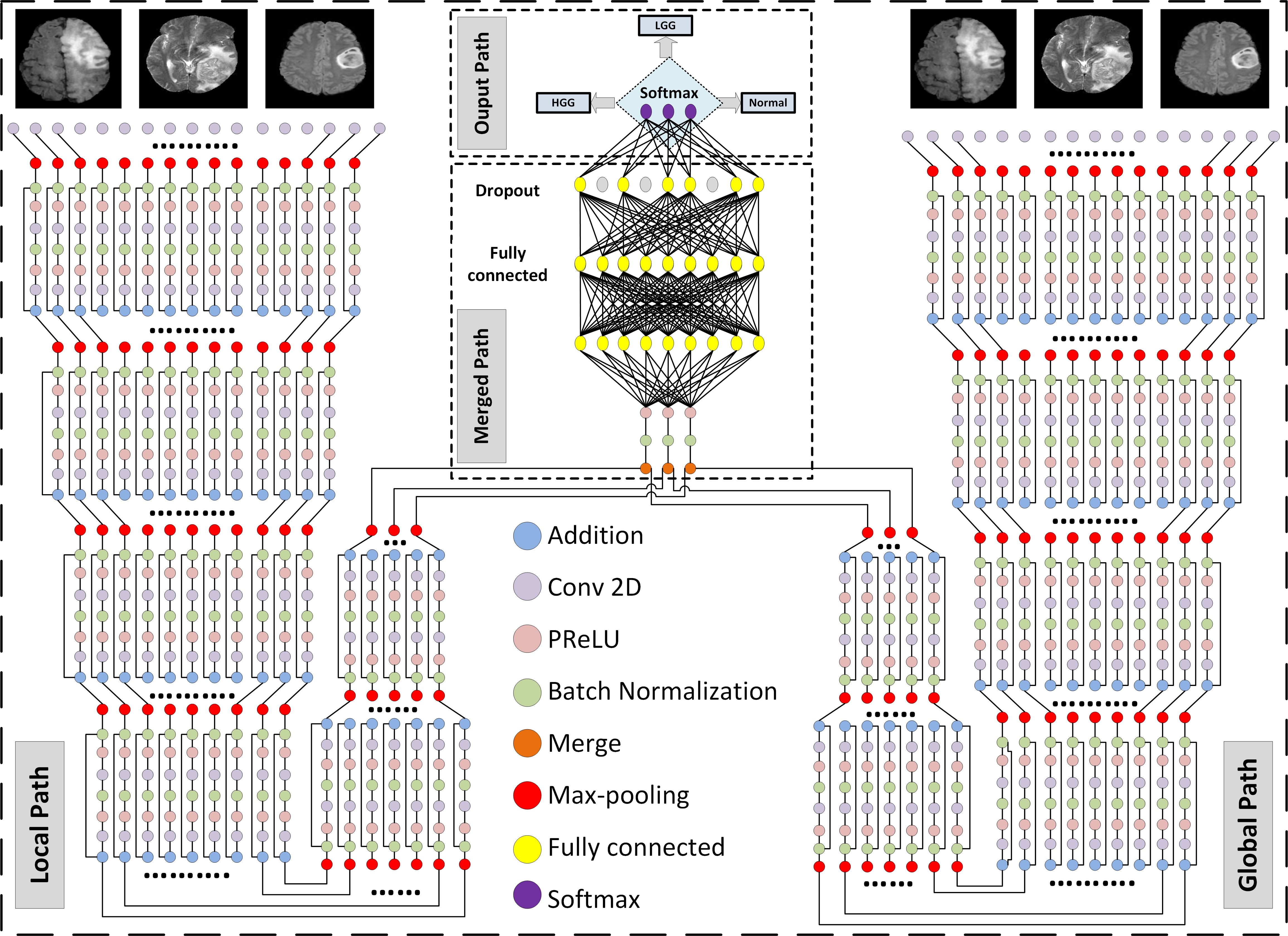

Supplement: Supplementary file 1 — Supplementary Information. [file 41598_2024_59566_MOESM1_ESM.zip › Figures/Figure-01.jpg]

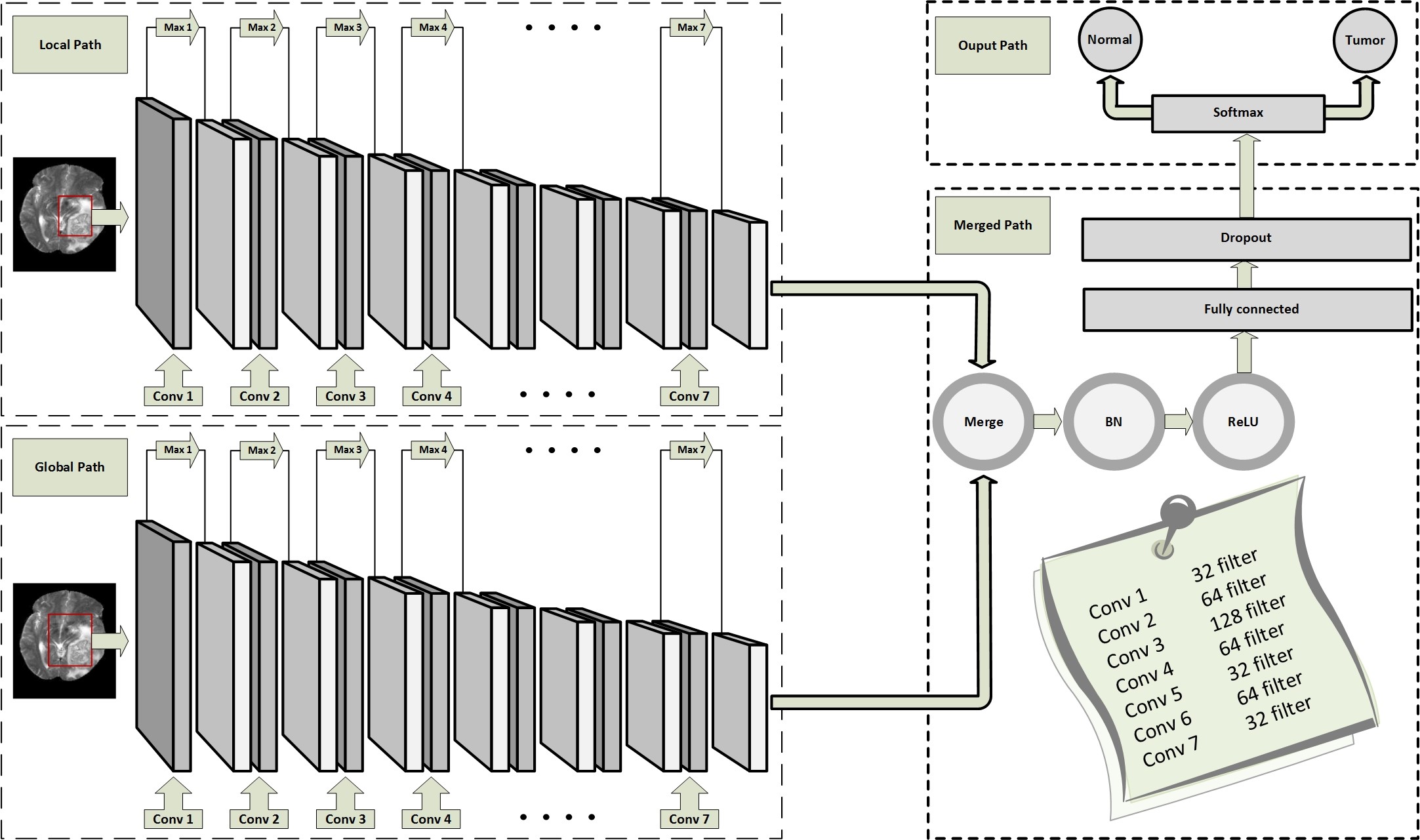

Supplement: Supplementary file 1 — Supplementary Information. [file 41598_2024_59566_MOESM1_ESM.zip › Figures/Figure-01xxx.jpg]

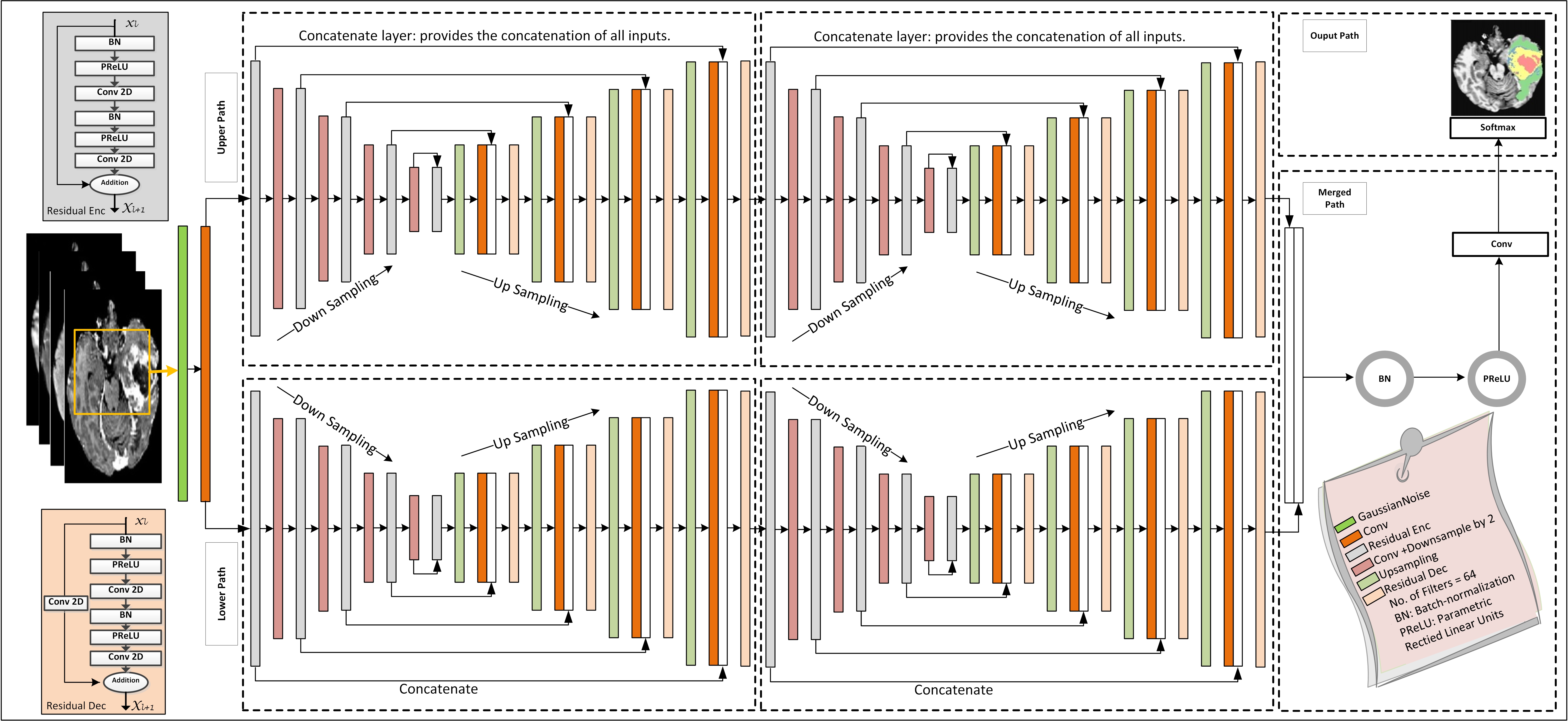

Supplement: Supplementary file 1 — Supplementary Information. [file 41598_2024_59566_MOESM1_ESM.zip › Figures/Figure-002.jpg]

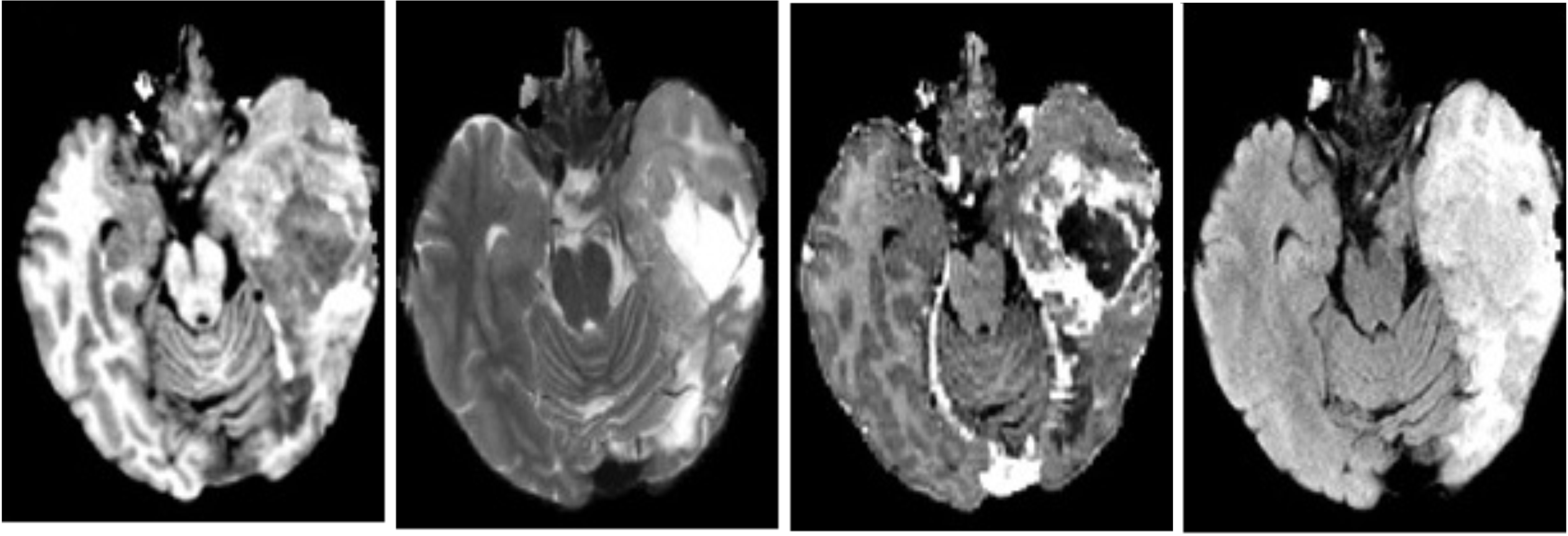

Supplement: Supplementary file 1 — Supplementary Information. [file 41598_2024_59566_MOESM1_ESM.zip › Figures/Preprocessing.jpg]
